# Targeted Therapies in the Most Common Advanced Solid Tumors, Drug Resistance, and Counteracting Progressive Micrometastatic Disease: The Next Frontier of Research

**DOI:** 10.1002/mco2.70373

**Published:** 2025-09-27

**Authors:** Andrea Nicolini, Paola Ferrari, Roberto Silvestri, Dario A. Bini

**Affiliations:** ^1^ Department of Oncology and New Technologies in Medicine University of Pisa Pisa Italy; ^2^ Unit of Oncology 1 Azienda Ospedaliera Universitaria Pisana Pisa Italy; ^3^ Department of Biology Genetic Unit University of Pisa Pisa Italy; ^4^ Department of Mathematics University of Pisa Pisa Italy

**Keywords:** solid tumors, drug resistance, micrometastatic disease, immune suppression, immune therapy, serum tumor markers

## Abstract

The era of targeted therapies has significantly advanced our understanding of cancer growth and metastasis. Intrinsic or acquired drug resistance remains a major challenge, rendering clinically overt metastatic disease incurable in most patients. This review first examines key clinical trials and their primary outcomes involving targeted therapies in the most common advanced solid tumors, along with the main mechanisms underlying drug resistance. Recently, micrometastatic disease has emerged as a novel focus of investigation aimed at definitively curing advanced solid tumors. Accordingly, this review explores the biology of micrometastases, current challenges in their detection and monitoring, and the main strategies proposed to prevent their progression. The potential roles of nanotechnology and artificial intelligence‐driven predictive models are also discussed. Furthermore, we highlight specific characteristics of micrometastatic disease that favor immune modulation, and we evaluate the effectiveness of an immunotherapy regimen that inhibits immune suppression. The lead time provided by serum tumor markers, used experimentally to better track the progression of otherwise undetectable micrometastatic disease, also forms the mechanistic basis for a novel protocol we propose to prevent relapse in high‐risk cancer patients. This innovative protocol holds scientific relevance being supported by an appropriate mathematical model and ready for immediate application in clinical practice.

## Graphical abstract

In TME, undetectable DCCs (panel A) join with a low tumor burden along with low immune suppression, that favor successful immune manipulation through immune suppression inhibiting immune‐therapy. This moves the immune balance toward the immune response and likely makes more stable the dormant state of DCCs in the unstable metastatic niche and/or allows innate and adaptive immune response to kill the cancer cells. In clinically overt metastatic disease (panel B), higher tumor burden and immune suppression favor cancer cell proliferation and the arising of drug resistance. ↑ decreased IS; ↑ increased IS; ↑ further increased IS.

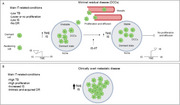

## 1 Introduction

“Targeted therapies” were originally conceived as a means to definitively cure metastatic cancer; however, the majority of patients ultimately succumb to disease progression due to the emergence of drug resistance. While tamoxifen is often regarded as an “ante litteram” targeted therapy, the advent of the “targeted therapy” or “precision medicine” era is commonly dated to 2001, when Slamon et al. [1] first reported the clinical utility of trastuzumab—a monoclonal antibody administered to patients with relapsed breast cancer overexpressing human epidermal growth factor receptor 2 (HER2). Around the same period, Hanahan and Weinberg [2] introduced their seminal framework on the hallmarks of cancer in 2000, and subsequently refined it in 2011 [3]. These conceptual advances significantly propelled investigations into the molecular signaling pathways and genetic and epigenetic aberrations that underpin cancer progression and dissemination [4]. Concurrently, numerous randomized clinical trials incorporating targeted therapies facilitated the translation of these molecular and genomic insights into clinical practice [4]. Mechanistically, targeted therapies act either by inhibiting oncogenic signaling pathways that drive tumor proliferation or by enhancing host antitumor immunity through immune modulation [5, 6, 7].

More recently, the prevention of clinically manifest metastatic disease has been proposed as an emerging therapeutic paradigm aimed at achieving definitive cancer cures. This review seeks to contribute to this evolving discourse by first summarizing the principal clinical trials evaluating targeted therapies in the most prevalent advanced solid tumors, with emphasis on their key outcomes. In addition, the review addresses the principal mechanisms underlying therapeutic resistance. Although a substantial proportion of patients initially respond to endocrine therapies or cytotoxic chemotherapy, resistance—either intrinsic (primary) or acquired—inevitably ensues, leading to treatment failure.

Drug resistance can arise following several mechanisms; for example, mutations within the target sequence, hampering the drug‐target binding, are widely reported as well as the activation of alternative pathways that can bypass the effect of the target inhibition and that can depend on activating mutation or overexpression of other oncogenic proteins. Particular attention is given to the critical role of the tumor microenvironment (TME) while mechanisms involving epigenetic dysregulation and less commonly reported pathways are also considered.

Subsequently, this review outlines the biological rationale for targeting micrometastatic disease as a strategic approach for the definitive treatment of high‐risk patients with solid tumors. The potential contributions of nanotechnology and artificial intelligence (AI)‐driven predictive models, along with their projected development in the coming decade, are also explored.

Within the context of emerging therapeutic strategies aimed at preventing micrometastatic disease progression, a novel immune suppression‐inhibiting immunotherapy (IS‐IIT) schedule is proposed, with particular applicability to disseminated cancer cells (DCCs). Finally, the review discusses the kinetics of DCC proliferation, as inferred through serum tumor marker lead time (LT), and presents the mechanistic rationale underlying a protocol designed to eradicate DCCs in patients with solid tumors at high risk of relapse.

## 2 Targeted Therapies in Common Advanced Solid Tumors

In this section, we review the characteristics of the principal clinical trials conducted with targeted therapies in the most common advanced solid tumors. Lung, breast, gastrointestinal, prostate, and ovarian cancers are discussed in separate subsections.

### 2.1 Non‐Small Cell Lung Cancer

Lung cancer is a heterogeneous disease, which poses challenges for therapeutic decision‐making. In cases of advanced non‐small cell lung cancer (NSCLC), testing for predictive biomarkers is recommended, including epidermal growth factor receptor (EGFR), anaplastic lymphoma kinase (ALK), ROS proto‐oncogene 1 (ROS1), proto‐oncogene B‐raf (BRAF) V600E, neurotrophic tyrosine receptor kinase (NTRK) and RET fusions, MET exon 14 skipping alterations, and programmed death‐ligand 1 (PD‐L1) expression via immunohistochemistry (IHC) [8]. Additionally, emerging biomarkers such as EGFR exon 20 insertions, ERBB2 mutations, NRG1 fusions, Kirsten rat sarcoma viral oncogene homolog (KRAS) G12C, and tumor mutational burden are increasingly considered. In contrast, for lung squamous cell carcinomas (SCCs), PD‐L1 IHC is currently the only routinely recommended test [9]. As the number of actionable genomic alterations has grown, comprehensive next‐generation sequencing (NGS) assays—capable of evaluating multiple relevant biomarkers in a single analysis—have become increasingly advantageous [8, 9]. IHC and fluorescence in situ hybridization (FISH) may be used to complement NGS testing. Furthermore, the integration of small tissue biopsies and genotyping based on circulating cell‐free DNA (cfDNA) has become more common in routine clinical practice.

#### 2.1.1 Patients with Common (Exon 19 Deletion, Exon 21 L858R Mutation), Uncommon (S768I, L861Q, G719X Mutation) or Exon 20 Insertion EGFR Mutation‐Positive NSCLC

In NSCLC adenocarcinomas, more than 85% of common EGFR mutations confer sensitivity to EGFR‐tyrosine kinase inhibitors (TKIs), while the remaining ∼15% represent rare or uncommon EGFR mutations (Table 1) [10]. Osimertinib was the first third‐generation EGFR‐TKI approved for first‐line treatment in NSCLC patients harboring common EGFR mutations, receiving regulatory approval in multiple countries, including those in the European Union [11] and the United States [12, 13]. Two pivotal studies supported its approval in both regions [14, 15] (Table 1).

**TABLE 1:** Characteristics and findings in principal clinical trials carried out with the molecular targeted therapies as first line treatment of advanced NSCLC.

					Clinical outcome					
		Arms			mPFS (mo.)		mOS (mo.)			
Clinical trial (phase)	Study population	St.g.	C	Therapeutic intervention	Median	Increase (mo.)	Median	Increase (mo.)	G3/4 AEs (%)	References
Retrospective analyses of data from multiple observational studies or clinical trials	Uncommon EGFRms (S7681, L861Q, G719X)	96 total^a^ (6–26 range)		Afat 40 mg/day*	9.2 (5.2–19.7 range)	NA	NA	NA	NA	[10]
		39 total^a^ (6–14 range)		Gefit 250 mg/day	5.45 (5.4–8.8 range)	NA NA	NA	NA	NA	[10]
		17 total^a^ (5–12 range)		Erlot 150 mg/day	6.55 (5–12 range)	NA NA	NA	NA	NA	[10]
		693 total^a1^ (1–294 range)		Afat 40 mg/day	10.8 (8.1–16.6 range)	NA	NA	NA	NA	[16]
FLAURA (III)	Common EGFR ms (exon 19d or exon 21 L858Rm)	279	277	Osimert vs. Gefit/Erlot (up to progression or toxicity)*	18.9 vs. 10.2 *p* < 0.001	8.7	38.6 vs. 31.8 *p* = 0.046	6.8	42 vs. 47	[14]
FLAURA 2 (III)		278	279	Osimert plus Pem plus CBDCA or CDDP vs. Osimert monotherapy	25.5 vs. 16.7 IA *p* < 0.01 29.4 vs. 19.9 BICR *p* < 0.01	8.8 9.5	NA	NA	64 vs. 27	[15]
PROFILE 1029 (III)	ALK rearrangement	104	103	Crizot vs. Pem + CBDCA or Pem + CDDP	11.1 vs. 6.8 *p* < 0.001	4.3	28.5 vs. 27.7	0.8	11.5	[24]
ASCEND‐4 (III)		189	187	Cerit vs. Pem + CBDCA or Pem + CDDP and Pem m	16.6 vs. 8.1 *p* < 0.00001	8.5	NA	NA	78 vs. 62	[25]
ALEX (III)		151	152	Crizot vs. Alect	11.1 vs. NR 10.4 vs. 25.7 IRCA	15.3 IRCA *p* < 0.001	NR vs. 57.4	NA	Serious 29 vs. 28	[26, 27]
Integrated analysis	ROS1 fusion positive	172 (total) 67 (1st line)		Entrectinib	16.8 17.7	NA	Immature 47.7	NA	Serious 14	[31]
NCT01336634 (II)	BRAF V600E	36^b^ vs. 37 or 28		Dab + Tram vs. PBC or ICI + PBC	10.2 vs. 4.2 (PBC) or 11.3	+6 (PBC) or −1.1 (ICI‐PBC)	17.3 vs. 9.7 or 18	+ 7.6 (PBC) or −0.7 (ICI‐PBC)	NA	[32]
NAVIGATE (II) NCT02122913 (I)	NTRK 1/2/3 gene fusion	20 total		Larotrec^c^	33.9	NA	40.7	NA	10	[36]
GEOMETRY mono‐1 (II)	MET ex 14 skipping mutation	97 total		Capimat^d and d1^	12.4^d^ 5.4^d1^	NA	NA	NA	67	[41, 42]
VISION (II)		99 total (evaluable)		Tepot^d and d1^	8.5	NA	NA	NA	28	[43]
LIBRETTO‐001 (I/II)	RET rearrangement	316 total		Selpercat 69^d^ + 247^d1^	22^d^ 24.9^d1^	NA	At 3 yrs 57.1%^d^ 59.5%^d1^	NA	38	[44, 45]
ARROW (I/II)		211 total		Pralset 75^d^ + 211^d1^	13^d^ 16.5^d1^	NA	At 1 yr 82%^d^ 72%^d1^	NA	20	[46, 47]
DESTINY‐Lung‐01 (II)	HER2 mutation	91 total		TDXd^e^	8.2	NA	17.8	NA	46	[48]
KEYNOTE‐024 (III)	PD‐L1+	154	151	Pembro vs. PBC	10.3 vs. 6 *p* < 0.001	4.3	At 6 mo. 80.2 vs. 72.4%	NA	26 vs. 53.3	[51]
KEYNOTE‐407* (III)		278^f^	281^f^	Pembro + Ct vs. Ct	6.4 vs. 4.8 *p* < 0.001	1.6	15.9 vs. 13.2 *p* < 0.001	2.7	69.8 vs. 68.2	[54]
KEYNOTE‐189** (III)		410	206	Pembro + Pem and PBC vs. Pem and PBC	8.8 vs. 4.9 *p* 0.001	3.9	At 1 yr 69.2 vs. 49.4%	NA	67.2 vs. 65.8	[59]
EMPOWER‐Lung‐1 (III)		283	280	Cemip vs. PBC	8.2 vs. 5.7 *p* < 0.0001	2.5	17.9 vs. 14.2 *p* = 0.0002	3.7	28 vs. 39	[60]
IMPOWER‐110 (III)		107^g^ (wt)	98^g^ (wt)	Atezo vs. PBC	8.1 vs. 5	3.1	20.2 vs. 13.1	7.1	30.1 vs. 52.5	[63]
IMPOWER‐150 (III)		356 ^h^ (wt)	356^h^ (wt)	ABCP vs. BCP	8.3 vs. 6.8 *p* < 0.001)	1.5	19.2 vs. 14.7 *p* = 0.02	4.5	NA	[64]
CHECKMATE‐9LA (III)		361^i^	358^i^	Nivo + Ipi + PBC (2 cycles) vs. PBC (4 cycles)	NA	NA	15.6 vs. 10.9	4.7	24 vs. 27	[65]

Abbreviations: EGFRms: epidermal growth factor receptor mutations; d: deletion; m: mutation; St.g.: study group; C: controls; mPFS: median PFS; mOS: median OS; mo.: months; AEs: adverse events; Osimert: osimertinib; Pem: pemetrexed; Pem m: Pem maintenance; CBDCA: carboplatin; CDDP: cisplatin; IA: independent assessment; BICR: blinded independent central review; Gefit: gefitinib; Erlot: erlotinib; Afat: afatinib; NA: not available; a: patients assessed in the different studies or clinical trials, total number and range; in some trials a comparison among different mutation groups that has not been reported was carried out; a^1^: data were available for 272 EGFR TKI (tyrosine kinase inhibitor) naïve patients; Crizot: crizotinib; Cerit: ceritinib; Alect: alectinib; NR: not reached; IRCA: independent review committee assessment; b: 36 patients from phase II trial compared with 35 or 28 patients from real world standard of care in retrospective analysis; Dab: dabrafenib; Tram: trametinib; Larotrect: larotrectinib; c: 95% of the studied population had received previous chemotherapy with or without other targeted therapy; Capimat: capimatinib; d: not previously treated; d1: with one or more lines of systemic therapy; Tepot: tepotinib; Selpercat: selpercatinib; Pralset: pralsetinib; TDXd: trastuzumab‐deruxtecan; e: previously treated patients (from zero to 7 lines of therapy); 95% had received PBC and 60% PD1 or PD‐L1 inhibitors; f: most with positive PD‐L1 cancer cells; Ct: chemotherapy; Pembro: pembrolizumab; *in squamous NSCLC; WT: wild‐type population excluded patients with EGFR or ALK genetic aberrations; Cemip: cemiplimab; **PD‐L1 at least 50%; g: high PD‐L1 expression in cancer or immune cells; H: PD‐L1 expression evaluated in cancer or immune cells could be low or negative; Atezo: atezolizumab; ABCP: atezolizumab plus bevacizumab plus carboplatin plus paclitaxel; BCP: bevacizumab plus carboplatin plus paclitaxel; I: randomization was stratified by PD‐L1 expression; Nivo: nivolumab; Ipi: ipilimumab (also see text).

Across 10 studies, uncommon EGFR mutations—including exon 20 insertions within the tyrosine kinase domain—accounted for approximately 1–18% of all EGFR mutations. Afatinib, a second‐generation EGFR‐TKI, is the preferred first‐line option for NSCLC with certain uncommon mutations [13], and its efficacy has been evaluated in two retrospective analyses [10, 16]. More recently, a phase II nonrandomized clinical trial demonstrated that osimertinib may be an effective treatment for previously untreated or relapsed NSCLC patients with uncommon EGFR mutations, excluding those with exon 20 insertions [17].

Amivantamab‐vmjw, a bispecific antibody targeting EGFR and MET receptors, initially received approval for the treatment of advanced NSCLC with EGFR exon 20 insertion mutations in patients who had progressed following platinum‐based chemotherapy (PBC) [18]. In March 2024, the United States Food and Drug Administration (US FDA) approved the combination of amivantamab‐vmjw with carboplatin and pemetrexed as first‐line therapy for the same patient population. The approval was based on data from the PAPILLON trial [19], which showed that the addition of amivantamab‐vmjw to chemotherapy significantly improved median progression‐free survival (PFS) compared with chemotherapy alone (11.4 vs. 6.7 months; *p* < 0.0001), overall survival (OS) data remain immature.

#### 2.1.2 Patients with a KRAS G12C Mutation or Anaplastic Lymphoma Receptor Tyrosine Kinase Gene (ALK) Rearrangement or ROS1 Rearrangement

In May 2021, based on the results of the CodeBreaK 100 study, the US FDA granted accelerated approval to sotorasib for the treatment of patients with advanced NSCLC harboring the KRAS G12C mutation, following at least one prior systemic therapy (Table 1). Sotorasib demonstrated a PFS of 6.3 months, an OS of 12.5 months, and a 2‐year OS rate of 33%. Common adverse events included diarrhea (30%) and elevated alanine and aspartate aminotransferase levels (18% each) [20]. Approximately 3–5% of NSCLC patients have rearrangements involving the ALK tyrosine kinase receptor [21], with similar prevalence in East Asian and non‐Asian populations [22]. In the phase III randomized PROFILE 1014 trial, crizotinib, administered to treatment‐naive patients with ALK‐positive advanced nonsquamous NSCLC, demonstrated superior efficacy compared with first‐line chemotherapy [23]. Additional key studies involving ALK inhibitors—including crizotinib, ceritinib, and alectinib—are summarized in Table 1 [24, 25, 26, 27]. In 1–2% of NSCLC cases, the ROS1 tyrosine kinase receptor, a member of the insulin receptor family, undergoes chromosomal rearrangements. The resulting ROS1 fusion genes drive oncogenic signaling through activation of multiple pathways, including mitogen‐activated protein kinase (MAPK)/extracellular signal‐regulated kinase (ERK), phosphoinositide 3‐kinase (PI3K), protein kinase B (AKT), mammalian target of rapamycin (mTOR), and Janus kinase (JAK)/signal transducer and activator of transcription (STAT), promoting cancer cell survival, proliferation, and growth. These fusions also confer constitutive, ligand‐independent catalytic activity [28]. Entrectinib, a potent ROS1 inhibitor with central nervous system (CNS) activity, has been evaluated across three phase I/II clinical trials [29, 30]. An extended follow‐up analysis (37.8 vs. 29.1 months) with an expanded patient cohort (172 vs. 168) further reinforced its clinical benefit [31]. As a result, entrectinib is currently recommended as a first‐line treatment option for patients with ROS1 fusion‐positive NSCLC, including those presenting with baseline CNS metastases.

#### 2.1.3 BRAFV600E Mutation or NTRK1/2/3 Gene Fusion or METex14 Skipping Mutation or RET Rearrangement‐Positive Patient

BRAF mutations are present in approximately 2–5% of lung adenocarcinomas, with 45–83% of these classified as the BRAFV600E subtype (Table 1). These mutations result in constitutive activation of the RAS–RAF–MEK–ERK signaling cascade and are more frequently observed in individuals with a history of smoking [32, 33]. Currently, PBC and/or immune checkpoint inhibitors (ICIs) represent standard treatment options for advanced NSCLC lacking EGFR or ALK alterations [34]. However, patients with BRAFV600E mutations tend to have lower response rates to PBC, and ICI response rates range from 24 to 35% in this subgroup [32, 33]. Dabrafenib, a BRAF inhibitor, in combination with trametinib, a MEK inhibitor, is the only approved targeted therapy for advanced BRAFV600E‐mutated NSCLC. The efficacy of this combination was demonstrated in a noncomparative phase II clinical trial [35].

Tropomyosin receptor kinase (TRK) proteins—TRKA, TRKB, and TRKC—are encoded by the *NTRK1*, *NTRK2*, and *NTRK3* genes, respectively. TRK fusions act as oncogenic drivers in fewer than 1% of NSCLC cases, predominantly in adenocarcinomas arising in nonsmokers or light smokers, often at younger ages. Larotrectinib, a CNS‐penetrant TRK inhibitor [36, 37, 38], is approved for the treatment of adult and pediatric patients with TRK fusion‐positive solid tumors [39], based on durable antitumor activity observed in a pooled analysis of three phase I/II trials [36], subsequently validated in a larger cohort [37]. The National Comprehensive Cancer Network (NCCN) currently recommends both larotrectinib and entrectinib as first‐line options for advanced NSCLC harboring *NTRK* fusions [40]. The *MET* proto‐oncogene, located on chromosome 7q21–q31, can be altered in NSCLC via exon 14 skipping mutations (observed in 3–4% of patients) or gene amplification (1–6%). Additionally, *RET* proto‐oncogene fusions occur in approximately 1–2% of NSCLC cases. Key clinical trials and efficacy data for MET inhibitors (capmatinib and tepotinib) and RET inhibitors (selpercatinib and pralsetinib) are summarized in Table 1 [41, 42, 43, 44, 45, 46, 47].

#### 2.1.4 ERBB2 Mutation‐Positive Patients and Patients with PD‐L1> or <1% and Negative for Actionable Molecular Biomarkers

Unlike breast and gastric cancers, human HER2‐mutant NSCLC—accounting for approximately 3% of nonsquamous NSCLC cases—is typically treated with chemotherapy or immunotherapy (Table 1). Trastuzumab deruxtecan, a humanized anti‐HER2 monoclonal antibody conjugated to a topoisomerase I inhibitor, has been evaluated in patients with metastatic HER2‐mutant NSCLC who were refractory to standard therapies [48].

The majority of NSCLC patients do not harbor actionable oncogenic drivers. Among these, approximately 23–28% exhibit PD‐L1 overexpression (≥50% of tumor cells) [49, 50]. In the KEYNOTE‐024 trial, previously untreated patients with advanced NSCLC, high PD‐L1 expression, and no EGFR or ALK alterations were randomized to receive either pembrolizumab (a programmed cell death protein 1 [PD‐1] inhibitor) or PBC at the investigator's discretion [51].

Squamous NSCLC comprises 20–30% of all NSCLC cases and generally has a poorer prognosis than nonsquamous subtypes. Due to the limited presence of targetable alterations, cytotoxic chemotherapy remains the standard treatment option for this group [52, 53, 54]. However, for patients with PD‐L1 expression >50% on tumor cells, first‐line treatment with a PD‐1 or PD‐L1 inhibitor in combination with chemotherapy has been shown to significantly enhance response rates and improve survival, likely due to the immunogenic effects of cytotoxic agents [55, 56, 57, 58, 59].

In the EMPOWER‐Lung 1 trial, cemiplimab monotherapy demonstrated a survival benefit over chemotherapy in patients with high PD‐L1 expression [60]. As a result, NCCN guidelines now recommend cemiplimab as a first‐line treatment option for this population [13]. The subsequent EMPOWER‐Lung 3 trial assessed cemiplimab in combination with platinum‐doublet chemotherapy, administered regardless of PD‐L1 expression, in both squamous and nonsquamous NSCLC [61]. Atezolizumab combined with platinum‐doublet chemotherapy has been approved exclusively for nonsquamous NSCLC [62, 63, 64]. Additionally, the combination of ipilimumab (anti–CTLA‐4) and nivolumab (anti‐PD‐1), with or without chemotherapy, has received approval for the treatment of advanced NSCLC across histologic subtypes [65, 66].

### 2.2 Breast Cancer

Recently, we addressed this issue in the context of advanced breast cancer [67]; therefore, the principal reported data on this cancer are summarized in Table 2 [1, 68, 69, 70, 71, 72, 73, 74, 75, 76, 77, 78, 79, 80, 81, 82, 83, 84, 85, 86, 87, 88].

**TABLE 2:** Characteristics and findings in principal clinical trials carried out with anti‐HER2 mAbs, ICIs, Akt inhibitors, poly‐ADB ribose polymerase (PARP) inhibitors, and CDK inhibitors as first line therapy in advanced breast cancer.

					Clinical outcome					
		Arms			mPFS (mo.)		mOS (mo.)			
Clinical trial (phase)	Study population	St.g.	C	Therapeutic intervention	Median	Increase (mo.)	Median	Increase (mo.)	G3/4 AEs (%)	References
Randomized (III)	HER2+	235	234	Trst + Ct^a^ vs. Ct^b^	7.4 vs. 4.6 *p* < 0.001	2.8	25.1 vs. 20.3 *p* < 0.001	4.8	27 or 13^a^ vs. 8 or 1^b^	[1]
Cleopatra (III)		402	406	Trst + Prtz + Dtx vs. Trst + PBO + Dtx	18.5 vs. 12.4 *p* < 0.001	6.1	57.1 vs. 40.8 *p* < 0.01	16.3	Similar in the 2 groups	[68, 69]
Keynote‐355 (III)	TNBC	566	281	Pembro + Ct^c^ Vs. PBO + Ct	9.7 vs. 5.6 *p* = 0.0012; 7.6 vs. 5.6 *p* = 0.0014; 7.5 vs. 5.6 *p* = ns (ITT)	4.1 2 1.9	23 vs. 16.1 *p* = 0.0019 (CPS > 10) 17.6 vs. 16 *p* = ns (CPS>1) 17.2 vs. 15.5 *p* = ns (ITT)	2.3 1.6 1.7	68.1 vs. 66.9	[70, 71]
Impassion 130 (III)		451	451	Atz + NabPtx vs. PBO + NabPtx	7.2 vs. 5.5 *p* = 0.002 (ITT) 7.5 vs. 5.5 *p* < 0.01 (PD‐L1+)	1.7 (ITT) 2 (PD‐L1+)	21 vs. 18.7 *p* = ns (ITT) 25.4 vs. 17.9 *p* = ns (PD‐L1+)	2.3 (ITT) 7.5 (PD‐L1+)	16.7 vs. 12.9	[72, 73]
Impassion 131 (III)		191	101	Atz + Ptx Vs. PBO + Ptx	6 vs. 5.7 *p* = ns (PD‐L1+)	0.3	22.1 vs. 28.3 *p* = ns (PD‐L1+)	−6.2	11 vs. 5	[74]
Lotus (II)		62	62	Ipatasertib + Ct vs. Ct	6.2 vs. 4.9	1.3	25.8 vs. 16.9	8.9	>15	[75, 76]
PAKT (II)		70	70	Capivesertib + Ct vs. Ct	5.9 vs. 4.2	1.7	19.1 vs. 12.6	6.5	>15	[77]
OlympiA‐D (III)	gBRCA mut, HER2−	205	97	Olaparib vs. Ct ^d^	7 vs. 4.2 *p* < 0.001	2.8	19.3 vs. 17.1 *p* = ns	2.1	36.6. vs. 50.5	[78, 79]
Embraca (III)		287	144	Talazoparib vs. Ct ^e^	8 vs. 5.6 *p* < 0.001	2.4	19.3 vs. 19.5 *p* = ns	−0.2	55 vs. 38 H 32 vs. 28 NH	[80, 81]
Bolero 2 (III)	HR+/HER2−	485	239	Eve + Exe ^f^ Vs. Exe ^f^	7.8 (11) vs. 3.2 (41)	4.6 (6.9)	31 vs. 26.6	4.4	42	[82, 83]
Monaleesa 2 (III)		324	324	Ribociclib + Let vs. Let	23.3 vs. 16	9.3	63.9 vs. 51.4	12.5	>10	[84, 85]
Monarch 3 (III)		328	165	Amebaciclib + NSAIs (Let/Ana) vs. NSAIs	28.2 vs. 14.8	13.4	NA	NA	58	[86]
Paloma 2 (III)		444	222	Palbociclib + Let vs. Let	27.6 vs. 14.5	13.1	53.9 vs. 51.2	2.7	>15	[87, 88]

Abbreviations: St.g.: study group; C: controls; mPFS: median progression‐free survival; mOS: median overall survival; mPFS: median PFS; mOS: median OS; mo.: months; AEs: adverse events; Trst: trastuzumab; Ct: chemotherapy; a: doxorubicin (Dox) and cyclophosphamide (Cy) (143) or paclitaxel (Ptx) (92); b: Dox (epirubicin in 36) and Cy (138) or Ptx (96); Prtz: pertuzumab; Dtx: docetaxel; PBO: placebo; Pembro: pembrolizumab; c: nabPtx or Ptx or gemcitabine (Gem) + carboplatin (CBDCA); ITT: intent to treat; TNBC: triple‐negative breast cancer; Atz: atezolizumab; d: capecitabine, eribulin or vinorelbine; e: capecitabine, eribulin, Gem or vinorelbine; gBRCAmut: germline BRCA1/2 gene mutation; H: hematologic; NH: not hematologic; Eve: everolimus; Exe: exemestane; f: with recurrence/progression during or after nonsteroidal aromatase inhibitors (NSAIs); HR+: hormone receptor positive; Let: letrozole; Ana: anastrozole; NA: not available; PD‐L1: programmed death ligand 1; CPS: combined positive score; NA: not available (also see text).

### 2.3 Gastrointestinal Cancer

In frequency order it includes colorectal and gastric cancers.

#### 2.3.1 Colorectal Cancer

Colorectal cancer (CRC) is currently the third most commonly diagnosed cancer and the second leading cause of cancer‐related mortality worldwide [89].

Cetuximab and panitumumab—both anti‐EGFR monoclonal antibodies—are commonly used as first‐line therapy in metastatic CRC patients who are *RAS* and *BRAF* wild‐type and HER2‐positive (defined as IHC 3+ or IHC 2+ with FISH positivity) [90]. Trastuzumab deruxtecan has demonstrated promising activity in HER2‐expressing CRC, while the combination of pertuzumab and trastuzumab has shown efficacy in HER2‐amplified, heavily pretreated, relapsed CRC patients, as reported in two separate phase II clinical trials [91, 92]. Antivascular endothelial growth factor (VEGF) or anti‐VEGF receptor (VEGFR) agents are also commonly used in both first‐ and second‐line treatment of unselected CRC populations [93].

In pretreated patients with *BRAFV600E* mutations, the triplet regimen of encorafenib, binimetinib, and cetuximab demonstrated superior efficacy compared with doublet therapy (encorafenib plus cetuximab) and standard treatment in the control arm [94]. Immunotherapies such as pembrolizumab or nivolumab (PD‐1 inhibitors) are typically used in relapsed or pretreated CRC patients exhibiting mismatch repair deficiency (dMMR) and/or high microsatellite instability (MSI‐H) [95, 96, 97]. The major clinical trials evaluating ICIs, bevacizumab, and anti‐EGFR monoclonal antibodies as first‐line therapies in advanced CRC are summarized in Table 3 [98, 99, 100, 101, 102, 103, 104, 105].

**TABLE 3:** Characteristics and findings in principal clinical trials carried out with ICIs or bevacizumab or anti‐EGFR mAbs as first line therapy in advanced colorectal cancer (aCRC).

					Clinical outcome					
		Arms			mPFS (mo.)		mOS (mo.)			
Clinical trial (phase)	Study population	St.g.	C	Therapeutic intervention	Median	Increase (mo.)	Median	Increase (mo.)	G3/4 AEs (%)	References
Keynote 177 (III)	MSI‐H or dMMR	153	157	Pembro vs. Ct^a^ with or without Beva or Cet	16.5 vs. 8.2 *p* = 0.0002	8.3	NA^b^ 13.7 Vs. 10.8	NA^b^ 2.9	22 vs. 66 (treatment‐related)	[98]
The BEAT Study (open‐label, observational)	Unresectable mCRC	300 503 552 346		5FU‐LV + Beva Folfiri + Beva Folfox + Beva Xelox + Beva	8.6 11.6 11.3 10.8	NA	18 23.7 25.9 23	NA	17 13.9 11.9 16.1	[99]
TRIBE Study (III)	mCRC BRAF WT KRAS WT or mutated	252	254	Folfoxiri vs. Folfoxiri + Beva or Folfiri + Beva	12.1 vs. 9.7 *p* = 0.003	2.4	31 vs. 25.8 *p* = 0.054	5.2	94.5 vs. 64.5 (c)	[100]
MRC COIN Trial (III)	aCRC KRAS WT	362	367	Ct^d^ + Cet vs. Ct^d^	8.6 vs. 8.6 *p* = 0.60)	0	17 vs. 17.9 *p* = 0.67	−0.9	31 vs. 3.8 skin 27 vs. 18 GI	[101]
THE NORDIC‐VII Study (III)	mCRC	ITT (total, *n* = 566)		Flox alone vs. cFlox + Cet or iFlox + Cet	7.9 8.3 7.3 *p* = ns	NA	20.4 19.7 20.3 *p* = ns	NA	22 and 29 (pts treated with CET) vs. 1 *p* < 0.01 Skin toxicity/rash	[102]
		185	194 187							
		KRAS WT 304 vs. 194 mut			8 vs. 8.1 *p *= ns	0.1	21 vs. 20.5 *p* = ns	0.5	NA	
		BRAF WT 402 vs. 55 mut			8.3 vs. 5.1 *p* = 0.001	3.2	22 vs. 9.5 *p* < 0.001	12.5	NA	
FIRE‐3 (III)	mCRC KRAS (exon 2) WT	297	295	Folfiri + Cet vs. Folfiri + Beva	10 vs. 10.3 *p* = 0.55	−0.3	28.7 vs. 25 *p* = 0.017	3.7	25 vs. 21 HT 26 vs. 2 SR 11 vs. 14 D	[103]
PRIME Study (III)	mCRC KRAS WT	325	331	Pan + Folfox‐4 vs. Folfox‐4	9.5 vs. 8 *p* = 0.02	1.5	23.9 vs. 19.7 *p* = 0.72	4.2	Commonly comparable but those associated with anti‐EGFR therapy	[104]
	mCRC KRAS mut	221	219	Pan + Folfox‐4 vs. Folfox‐4	7.3 vs. 8.8 *p* = 0.02	−1.5	15.5 vs. 19.3 *p* = 0.68)	−3.8		
NCT02394795 (III)	Unresectable mCRC RAS WT	411	412	mFolfox‐6 + Cet vs. mFolfox‐6 + Beva	12.2 vs. 11.4 *p* ns	0.8	36.2 vs. 31.3 *p* = 0.3	4.9	71.8 vs. 64.9	[105]

Abbreviations: St.g.: study group; C: controls; mPFS: median PFS; mOS: median OS; mo.: months; AEs: adverse events; MSI‐H: microsatellite instability high; dMMR: mismatch‐repair deficient; Pembro: pembrolizumab; Ct: chemotherapy; a: 5‐fluorouracil‐based therapy; Beva: bevacizumab; Cet: cetuximab; b: the estimated restricted mean survival after 24 months of follow‐up; mCRC: metastatic colorectal cancer; 5‐FU‐LV: 5‐fluorouracil leucovorin; Folfiri: irinotecan plus 5‐FU‐LV; Folfox: oxaliplatin plus 5‐FU‐LV; Xelox (or Capox): capecitabine plus oxaliplatin; c: excluding neutropenia that occurred in 50% of the study group vs. 20.3% in controls; d: oxaliplatin plus capecitabine or infusional 5‐FU‐LV plus Cet (St.g.) vs. the same Ct (C); GI: gastrointestinal toxic effects; Flox: oxaliplatin plus 5‐FU‐LV bolus; ITT; intent to treat; cFlox: Flox given continuously; iFlox: Flox given intermittently; WT: wild‐type; mut: mutated; HT: hematologic toxicity; SR: skin reaction; D: diarrhea; Pan: panitumumab; mFolfox‐6: modified Folfox‐6; NA: not available (also see text).

#### 2.3.2 Gastric Cancer

In Western countries, the incidence of gastric cancer has declined over the past several decades [106]. However, gastric cancer continues to have a poor prognosis, as it is frequently diagnosed at an advanced stage. In cases of advanced or metastatic disease, testing for HER2, PD‐L1, and claudin 18 isoform 2 (CLDN18.2) is recommended [107]. HER2 (*ERBB2*) amplification or overexpression is observed in approximately 15–20% of advanced gastric or gastroesophageal junction (GEJ) adenocarcinomas [108]. The phase III ToGA trial evaluated the efficacy and safety of trastuzumab in combination with chemotherapy (cisplatin plus fluorouracil or capecitabine) in patients with HER2‐positive advanced gastric adenocarcinoma [109]. The phase III KEYNOTE‐811 trial compared pembrolizumab versus placebo in combination with trastuzumab and chemotherapy (fluorouracil and cisplatin, or capecitabine and oxaliplatin) in previously untreated HER2‐positive advanced gastric adenocarcinoma. Median PFS and OS were improved in the pembrolizumab arm compared with placebo. Notably, patients with PD‐L1 combined positive score (CPS) ≥1 experienced longer median PFS with pembrolizumab versus placebo (10.8 vs. 7.2 months), whereas no benefit was observed in patients with PD‐L1 CPS <1 (9.5 vs. 9.6 months). As to median OS the PD‐L1 subgroup analysis followed a similar trend to the PFS analysis [110].

As a result, pembrolizumab was approved in combination with trastuzumab and fluoropyrimidine‐ and PBC as first‐line treatment for locally advanced, unresectable, or metastatic HER2‐positive gastric or GEJ adenocarcinoma. In 2023, this approval was restricted to patients with PD‐L1 CPS ≥1 [107]. For HER2‐negative advanced or metastatic gastric cancer, the phase III KEYNOTE‐859 trial evaluated pembrolizumab plus chemotherapy (fluorouracil with cisplatin or capecitabine with oxaliplatin) versus placebo plus chemotherapy in the first‐line setting. At a median follow‐up of 31 months, the pembrolizumab arm demonstrated improved OS and PFS compared with placebo [111, 112]. Based on these findings, pembrolizumab in combination with fluoropyrimidine‐ and PBC is recommended as a preferred first‐line treatment for patients with advanced HER2‐negative gastric cancer and PD‐L1 CPS ≥1, provided there is no prior exposure to ICIs or progression on ICI therapy [107]. Furthermore, pembrolizumab monotherapy or pembrolizumab combined with fluoropyrimidine and oxaliplatin is recommended as first‐line therapy for patients with MSI‐H or dMMR advanced gastric cancer, independent of PD‐L1 expression [113].

The phase III CheckMate‐649 trial evaluated nivolumab plus chemotherapy versus chemotherapy alone (capecitabine and oxaliplatin or mFOLFOX) as first‐line treatment for HER2‐negative advanced or metastatic gastric cancer [114, 115]. Nivolumab in combination with fluoropyrimidine‐ and oxaliplatin‐based chemotherapy is a preferred first‐line treatment for patients with HER2‐negative tumors and PD‐L1 CPS ≥1, in the absence of prior ICI therapy or progression on an ICI. This combination is considered a category 1, preferred option when PD‐L1 CPS ≥5 [107]. Approximately 3% of patients in CheckMate‐649 had MSI‐H tumors [114, 115], and these patients derived greater OS benefit from nivolumab plus chemotherapy compared with chemotherapy alone.

A subset of patients in CheckMate‐649 was also randomized to receive nivolumab plus ipilimumab or chemotherapy alone. At a minimum follow‐up of 24 months, patients with PD‐L1 CPS ≥5 and the overall study population did not show improved OS, PFS, or objective response rate with the nivolumab–ipilimumab combination versus chemotherapy alone [116]. However, in the MSI‐H subgroup, nivolumab plus ipilimumab was associated with improved median OS compared with chemotherapy (not reached vs. 10 months).

The phase III RATIONALE‐305 trial evaluated tislelizumab, a PD‐1 inhibitor, plus chemotherapy (investigator's choice of capecitabine and oxaliplatin or fluorouracil and cisplatin) in the first‐line setting for unresectable, HER2‐negative, locally advanced or metastatic gastric cancer [117]. Based on these findings, tislelizumab in combination with fluoropyrimidine (fluorouracil or capecitabine) and platinum (cisplatin or oxaliplatin) is recommended as first‐line treatment in patients with PD‐L1 CPS ≥1, provided they have received no prior ICI therapy or have not progressed on ICI. For patients with CPS ≥5, this is considered a category 1, preferred option [107].

Zolbetuximab, a monoclonal antibody targeting CLDN18.2, has recently been approved in combination with fluoropyrimidine‐ and PBC for patients with HER2‐negative, CLDN18.2‐positive advanced or metastatic gastric cancer. This combination showed a significant survival benefit in two phase III trials conducted in treatment‐naïve patients with HER2‐negative, CLDN18.2‐positive, unresectable locally advanced or metastatic disease. The SPOTLIGHT trial evaluated zolbetuximab plus mFOLFOX6 versus placebo plus mFOLFOX6 [118], while the GLOW trial evaluated zolbetuximab plus CAPOX versus placebo plus CAPOX [119]. Based on these results, fluoropyrimidine‐ and oxaliplatin‐based chemotherapy in combination with zolbetuximab is a category 1, preferred first‐line treatment option for patients with unresectable, locally advanced, recurrent, or metastatic gastric cancer that is HER2‐negative and CLDN18.2‐positive [107].

The main clinical trials involving anti‐HER2 monoclonal antibodies, ICIs, and anti‐CLDN18.2 antibodies as first‐line therapies in advanced gastric cancer are summarized in Table 4.

**TABLE 4:** Characteristics and findings in principal clinical trials carried out with anti‐HER2 mAbs, ICIs, and anti‐CLDN18.2 mAbs as first line therapy in advanced gastric cancer.

					Clinical outcome					
		Arms			mPFS (mo.)		mOS (mo.)			
Clinical trial (phase)	Study population	St.g.	C	Therapeutic intervention	Median	Increase (mo.)	Median	Increase (mo.)	G3/4 AEs (%)	References
ToGA (III)	HER2+	298	296	Trastuzumab plus Ct vs. Ct	6.7 vs. 5.5 *p* = 0.0002	1.2	13.8 vs. 11, *p* = 0.046	2.8	68 vs. 68	[109]
KEYNOTE‐811 (III)	HER2+	350	348	Pembrolizumab plus trastuzumab plus Ct vs. trastuzumab plus Ct	10.0 vs. 8.1 (*p* = 0.0002)	1.9	20.0 vs. 16.9, *p* = 0.084	3.1	58 vs. 51	[110]
KEYNOTE‐859 (III)	HER2− and PD‐L1 CPS ≥1 HER2− and PD‐L1 CPS ≥10	790	789	Pembrolizumab plus Ct vs. placebo plus Ct	6.9 vs. 5.6, *p* < 0.0001 8.1 vs. 5.6, *p* < 0.0001	1.3 2.5	13 vs. 11.4, p < 0.0001 15.7 vs. 11.8, *p* < 0.0001	1.6 3.9	“Serious” 23 vs. 19	[111]
Checkmate‐649 (III)	HER2− overall population HER2− and PD‐L1 CPS ≥1 HER2− and PD‐L1 CPS ≥5	789 641 473	792 655 482	Nivolumab plus Ct vs. placebo plus Ct	7.7 vs. 6.9 7.5 vs. 6.9 7.7 vs. 6.05 *p* < 0.0001	0.8 0.6 1.65	13.8 vs. 11.6 *p* 0.00002 14 vs. 11.3, *p* < 0.0001 14.4 vs. 11.1, *p* < 0.0001	2.2 2.7 3.3	59 vs. 44	[114]
RATIONALE‐305 (III)	HER2− (overall population) HER2− and PD‐L1 TAP score ≥5%	501 274	496 272	Tislelizumab plus Ct vs. placebo plus Ct	6.9 vs. 6.2 7.2 vs. 5.9 *p* = 0.001	0.7 1.3	15.0 vs. 12.9 *p* = 0.001 17.2 vs. 12.6 *p* = 0.006	2.1 4.6	54 vs. 50	[117]
SPOTLIGHT (III)	HER2−, CLDN18.2+	283	282	Zolbetuximab plus Ct vs. placebo plus Ct	10.61 vs. 8.67 *p* = 0.0066	1.9	18.23 vs. 15.54 *p* = 0.0053	2.7	87 vs. 78	[118]
GLOW (III)	HER2−, CLDN18.2+	254	253	Zolbetuximab plus Ct vs. placebo plus Ct	8.21 vs. 6.8, *p* = 0.0007	1.4	14.39 vs. 12.16, *p* = 0.0118	2.2	72.8 vs. 69.9	[119]

Abbreviations: St.g.: study group; C: controls; mPFS: median PSF; mOS: median OS; mo.: months; AEs: adverse events; CLDN18.2: claudin 18 isoform 2; Ct: chemotherapy; PD‐L1: programmed death ligand 1; CPS: combined positive score; TAP: tumor area positivity (also see text).

### 2.4 Prostate Cancer

According to the World Health Organization, prostate cancer is the third most common cancer and the second most frequently diagnosed cancer among men worldwide as of 2020 [89, 120]. Once prostate cancer progresses to the metastatic stage, its prognosis worsens significantly, with a 5‐year survival rate of approximately 30% [89, 120]. Over time, most patients develop resistance to first‐ and second‐line androgen deprivation therapies (ADT), progressing to a castration‐resistant prostate cancer (CRPC) phenotype, typically within 5 years of diagnosis [121]. Notably, approximately 25–30% of patients who initially undergo radical prostatectomy eventually progress to advanced disease and, subsequently, to androgen‐independent or CRPC status, which remains incurable with current standard therapies [122]. Reducing gonadal testosterone levels continues to be a cornerstone of treatment for advanced prostate cancer, as the majority of tumors express androgen receptors (AR), underscoring the central role of androgen signaling in prostate cancer pathogenesis [123, 124].

CRPC frequently emerges through increased AR signaling; however, some tumors evolve toward AR independence due to genetic and epigenetic alterations and hormonal changes that drive cellular plasticity. Over the past two decades, treatment for both metastatic hormone‐sensitive (mHSPC) and metastatic CRPC (mCRPC) has shifted toward genomically guided and novel targeted therapies [125, 126].

In mHSPC, ADT remains the foundational first‐line therapy [127, 128]. When ADT is limited to gonadotropin‐releasing hormone analogs, second‐line hormonal manipulation—such as the addition of steroidal antiandrogens like bicalutamide—is often employed for more complete androgen blockade [129]. In the early 2000s, mitoxantrone became the first cytotoxic chemotherapy approved by the US FDA for mCRPC [130]. It was later supplanted by taxane‐based therapies following the TAX327 and SWOG 99‐16 phase III trials, which demonstrated median OS improvements of 1.9 and 2.4 months, respectively, compared with mitoxantrone [131, 132]. The 2015 CHAARTED trial expanded the use of docetaxel to patients with mHSPC [133], while the subsequent approval of cabazitaxel by the US FDA and European Medicines Agency provided an effective option for docetaxel‐refractory mCRPC [134, 135].

Androgen receptor signaling inhibitors (ARSIs), poly(ADP‐ribose) polymerase inhibitors (PARPis), and immunotherapy represent emerging classes of targeted agents for defined molecular subsets. Despite advances in ADT, resistance often develops due to molecular alterations in AR signaling and the activation of AR‐driven transcriptional networks [136]. Abiraterone acetate and enzalutamide are next‐generation ARSIs designed to achieve maximal androgen blockade either by inhibiting androgen synthesis (abiraterone) or by antagonizing AR function (enzalutamide). Abiraterone acetate, a selective CYP17A1 inhibitor, demonstrated clinical efficacy in two phase III trials—COU‐AA‐301 and COU‐AA‐302—conducted in postdocetaxel and chemotherapy‐naive mCRPC patients, respectively [137, 138, 139]. Its benefit in high‐risk mHSPC was also demonstrated in the LATITUDE trial [140, 141] and corroborated by the abiraterone arm of the phase III STAMPEDE trial [142].

Enzalutamide functions by inhibiting AR binding of androgens, their nuclear translocation, and DNA interaction, even in tumors overexpressing AR. It received US FDA approval for mCRPC based on outcomes from the AFFIRM (postdocetaxel) and PREVAIL (chemotherapy‐naive) phase III trials [143, 144]. Subsequently, enzalutamide was evaluated in mHSPC in the ARCHES and ENZAMET phase III trials [145, 146, 147, 148]. In ARCHES, enzalutamide plus ongoing ADT was compared with ADT plus placebo. In ENZAMET, enzalutamide plus ADT was compared with first‐generation steroidal antiandrogens plus ADT. Positive results from both trials led the US FDA to expand enzalutamide's indication to mHSPC in August 2019 [149, 150].

To further extend the treatment landscape in metastatic prostate cancer, research has increasingly focused on tumor genomic profiling. PARPis have been evaluated for patients with alterations in DNA damage repair (DDR) and/or homologous recombination repair (HRR) genes [151]. In the phase III PROfound trial, olaparib significantly improved PFS compared with enzalutamide or abiraterone in mCRPC patients with DDR/HRR mutations who had progressed on ARSIs [152, 153, 154]. An exploratory analysis in cohort A of the PROfound trial (patients with *BRCA* or *ATM* alterations detected via circulating tumor DNA [ctDNA]) confirmed prolonged median radiographic PFS in the olaparib arm, supporting the clinical utility of ctDNA as an alternative to tissue‐based genotyping for *BRCA/ATM* alterations [155].

Two additional phase III trials—TALAPRO‐2 and KEYLYNK‐010—focused on mCRPC populations. TALAPRO‐2 evaluated first‐line talazoparib plus enzalutamide versus placebo plus enzalutamide, including both HRR‐deficient patients and an unselected cohort. The rationale for the trial was based on preclinical evidence of crosstalk between AR and PARP pathways, supporting dual inhibition [156, 157]. KEYLYNK‐010 enrolled previously treated, biGnRHomarker‐unselected mCRPC patients and compared pembrolizumab plus olaparib to a next‐generation hormonal agent [158]. Given that DDR deficiencies may increase tumor mutational burden and neoantigen load—thereby enhancing tumor‐infiltrating lymphocyte (TIL) recruitment—there is a growing interest in investigating ICIs in mCRPC.

The principal clinical trials involving ARSIs and PARPis, alone or in combination, for both mCRPC and mHSPC are summarized in Table 5.

**TABLE 5:** Characteristics and findings in principal clinical trials carried out in mCRPC and in mHSPC with ARSIs and/or PARPis alone or in association.

					Clinical outcome					
		Arms			mPFS(a) (mo.)		mOS (mo.)			
Clinical trial (phase)	Study population	St.g.	C	Therapeutic intervention	Median	Increase (mo.)	Median	Increase (mo.)	G3/4 AEs (%)	References
COU‐AA‐301 (III)	mCRPC after Dtx	797	398	AA plus PRD vs. PBO plus PRD	5.6 vs. 3.6	2	14.8 vs. 10.9	3.9	27 vs. 12(b) 10 vs. 1(c)	[137]
COU‐AA‐302 (III)	mCRPC Ct‐naive	546	542	AA plus PRD vs. PBO plus PRD	16.5 vs. 8.3	8.2	34.7 vs. 30.3	4.4	48 vs. 42	[138, 139]
LATITUDE (III)	Newly diagnosed mHSPC	597	602	AA plus PRD plus ADT vs. PBO plus PRD plus ADT	33 vs. 14.8	18.2	53.3 vs. 36.5	16.8	63 vs. 48	[140, 141]
AFFIRM (III)	mHSPC after 1–2 Ct lines	800	399	ENZA vs. PBO	8.3 vs. 2.9	5.4	18.4 vs. 13.6	4.8	45 vs. 53	[143]
PREVAIL (III)	mPC progressing despite ADT and before Ct	872	845	ENZA vs. PBO	Not reached vs. 3.9	NA	32.4 vs. 30.2 36 vs. 31	2.2 5	43 vs. 37	[144]
ARCHES (III)	mHSPC	572	574	ENZA plus ADT vs. PBO plus ADT	Not reached vs. 19	NA	NA	NA Death risk ‐34% in St.g. vs. C	39.2 vs. 27.9	[145, 147]
ENZAMET (III)	mHSPC	563	562	ENZA plus ADT vs. SNSAA plus ADT	68 vs. 41% (event‐free survival)	+27%	NA 67 vs. 57% (5‐years OS)	NA +10%	49 vs. 35 (G3) 7 vs. 7 (G4)	[146]
MAGNITUDE (III)	mCRPC HRR+	212(3)	211	Niraparib plus AA plus PRD vs. PBO plus AA plus PRD	16.7 vs. 13.7	3	NA	NA	57 vs. 43 (G3) 15 vs. 6 (G4)	[151]
	mCRPC BRCA1/2 mut	113	112		19.5 vs. 10.9	8.6	46% decrease in risk of death in St.g. vs. C		NA	
PROFOUND (III)	mCRPC progressing after AA or ENZA	256 (1)	131	Olaparib vs. AA plus PRD or ENZA	5.8 vs. 3.5	2.3	17.5 vs. 14.3	3.2	51 vs. 38	[152]
	Cohort A ctDNA subgroup	73(2)	38		7.39 vs. 3.53	3.86	17.5 vs. 13.5	4	NA	[155]
PROpel (III)	1‐line mCRPC	399	397	Olaparib plus AA plus PRD vs. AA plus PRD	24.8 vs. 16.6	8.2	NA	NA	47 vs. 38 (G>3)	[153]
					NA	NA	42.1 vs. 34.7	7.4	NA	[154]
TALAPRO‐2 (III)	1‐line mCRPC HRR deficient with and without HRR	200	199	Talazoparib plus ENZA vs. ENZA	Not reached vs. 13.8	NA	NA	NA	68 vs. 40 (G>3)	[156]
		395	398		NA	NA	30.8 vs. 25 TTD in GHS	5.8		[157]
KEYLYNK‐010 (III)	Pretreated, biomarker‐unselected mCRPC	529	264	Pembro plus olaparib vs. NHA (AA or ENZA)	4.4 vs. 4.2	0.2	15.8 vs. 14.6	1.2	34 vs. 9 (G>3)	[158]

Abbreviations: St.g.: study group; C: controls; mPFS: median PFS; mOS: median OS; AEs: adverse events; ARSis: androgen receptor signaling inhibitors; Dtx: docetaxel; AA: abiraterone acetate; PRD: prednisone; PBO: placebo; a: radiographic progression‐free survival; b: liver function test alteration; c: hypertension; Ct: chemotherapy; mPC: metastatic prostate cancer; NA: not available; ENZA: enzalutamide; SNSAA: standard nonsteroidal antiandrogen; 1: includes cohort A (*n* = 245 pts) with at least one alteration in BRCA1/2 or ATM genes and cohort B (*n* = 142 pts) with alterations in any of 12 other prespecified genes; 2: patients showing BRCA/ATM alterations in circulating tumor DNA (CtDNA); 3: with/without BRCA1/2 alterations; TTD: time to deterioration; GHS: global health status; Pembro: pembrolizumab; NHA: next‐generation hormonal agent.

### 2.5 Ovarian Cancer

Among gynecologic cancers in developed countries, ovarian cancer is responsible for the highest number of annual deaths, with an estimated 12,810 deaths in the United States in 2022 alone [159]. High‐grade serous ovarian cancer is the most prevalent histologic subtype, and approximately 50% of cases exhibit homologous recombination deficiency (HRD), which accounts for the observed high sensitivity to PBC and PARPis [160]. Although most patients are diagnosed at an advanced stage, earlier detection is more feasible in the less common clear cell and endometrioid subtypes [159, 161, 162, 163]. The current standard of care for advanced ovarian cancer consists of primary or interval cytoreductive (debulking) surgery—often following neoadjuvant chemotherapy—combined with adjuvant PBC [164, 165]. Achieving maximal cytoreduction is crucial, as the volume of residual disease is the strongest inverse predictor of both PFS and OS [166]. Bevacizumab, a recombinant monoclonal antibody targeting VEGFR signaling, was evaluated in combination with chemotherapy in two large randomized trials: GOG‐0218 and ICON7. While improvements in PFS were observed, neither study demonstrated a significant OS benefit [167, 168, 169]. Consequently, NCCN guidelines consider maintenance therapy with bevacizumab an “option” rather than a standard recommendation.

Additional studies have evaluated oral antiangiogenic agents in the maintenance setting following initial therapy [170, 171]; however, these agents also failed to demonstrate meaningful improvements in OS [172, 173]. The primary clinical trials assessing the role of PARPis as maintenance therapy following first‐line treatment are summarized in Table 6 [174, 175, 176, 177, 178, 179, 180, 181, 182, 183]. The utility of secondary cytoreduction in recurrent ovarian cancer has been examined in several trials [184]. While secondary debulking was associated with improved PFS, long‐term OS outcomes did not support a survival benefit from PARPi use in patients with either HRD‐BRCA wild‐type or BRCA‐mutated recurrent ovarian cancer [185, 186]. In light of this, recent American Society of Clinical Oncology guidelines state: “PARP inhibitor monotherapy maintenance (second line or later) may be offered to epithelial ovarian cancer (EOC) patients who have not previously received a PARPi and who have responded to PBC, regardless of BRCA mutation status” [187]. The next section provides an in‐depth analysis of the primary mechanisms underlying treatment failure—including resistance to both conventional and targeted therapies.

**TABLE 6:** Characteristics and findings in principal clinical trials carried out with PARP inhibitors alone or with bevacizumab as maintenance therapy after first line chemotherapy (Ct) in advanced ovarian cancer.

					Clinical outcome					
		Arms			mPFS (mo.)		mOS (mo.)			
Clinical trial (phase)	Study population	St.g.	C	Therapeutic intervention	Median	Increase (mo.)	Median	Increase (mo.)	G3/4 AEs (%)	References
SOLO1/GOG‐3004 (III)	BRCA1/2m responsive to PBC	261	131	PBC 6 cycles (4–9 range) followed by Olaparib (St.g.) or PBO (C) 2 years	56 vs. 13.8 *p* < 0.001	42.2	Not reached vs. 75.2 *p* = 0.004 (n.s.)	NA	Anemia (21 vs. 15%) MDS/AML (1.5 vs. 0%)	[174, 175, 176]
PRIMA/ENGOT‐OV26/GOG‐3012 (III)	Regardless of BRCA mutation status, responsive to PBC	487	246	CR or PR to PBC followed by Niraparib (St.g.) or PBO (C) 3 years	BRCAm HRd 51.5 vs. 11.5; *p* < 0.001	40	At 4 years HRd alive 38 vs. 17% and OP 24 vs. 14%	NA	Thrombocytopenia, anemia, neutropenia significantly. higher in St.g.	[177, 178]
					BRCAwt HRd 19.4 vs. 10.4; *p* < 0.001	9				
ATHENA‐MONO/ENGOT‐OV45/GOG‐3020 (III)	Regardless of BRCA or HRd status responsive to PBC	427	111	Platinum‐doublet Ct followed by Rucaparib (St.g.) or PBO (C) 2 years	HRd 28.7 vs. 11.3; *p* = 0.004	17.4	NA	NA	Anemia (28.7 vs. 0%), neutropenia (14.6 vs. 0.9%)	[179]
					ITT 20.2 vs. 9.2; *p* < 0.001	11				
VELIA/GOG‐3005 (III)	Regardless of Ct response or biomarker status	383^a^ 382^b^	375	6 cycles CBDCA + Ptx plus Veliparib followed by PBO maintenance^a^ or Veliparib maintenance^b^ vs. the same Ct plus PBO followed by PBO maintenance (C)	BRCAm 34.7 vs. 22 (C) *p* < 0.001	12.7	NA	NA	Anemia 41^a^ and 38^b^ vs. 26 Thrombocytopenia 31^a^ and 28^b^ vs. 8	[180, 181]
					HRd/BRCA wt 23 vs. 20 (C); HRP 15 vs. 11.5(C);	3;3.5				
					ITT 23.5 vs. 17.3 (C) *p* < 0.001	6.2				
PAOLA‐1/ENGOT‐OV25	Regardless of surgical outcome or BRCA mutation status; in clinical response after P‐TBC plus Beva	537	269	Olaparib 2 years plus Beva 15 mo (St.g.). vs. PBO 2 years plus Beva 15 mo. (C)	HRd/BRCAm 37.2 vs. 17.7	19.5	HRd positive 5 years 65.5 vs. 48.4%	NA	n.p.m. 4.1 vs. 3; pneu/br. 1.3 vs. 0.7	[182, 183]
					HRd/BRCAwt 28.1 vs. 16.6	11.5				
					ITT 22.1 vs. 16.6; *p* < 0.001	5.5	ITT 56.5 vs. 51.6 *p* = 0.41	4.9		

Abbreviations: St.g.: study group; C: controls; mPFS: median PFS; mOS: median OS; mo.: months; AEs; adverse events; BRCA1/2m: breast cancer 1/2 gene mutated; NA: not available; MDS/AML: myelodysplasia/acute myeloid leukemia; P‐TCB: platinum‐taxane based chemotherapy; HRd: homologous recombination deficiency; HRP: homologous recombination proficiency; NED: no evidence of disease; CBDCA: carboplatin; Ptx: paclitaxel; PBO: placebo; a: veliparib combination arm (St.g.); b: veliparib throughout arm (St.g.); Beva: bevacizumab; BRCAm: BRCA mutated; BRCAwt: BRCA wild‐type; ITT: intent to treat; ns: not significant; OP: overall population; CR: complete response; PR: partial response; n.p.m.: new primary malignancy; pneu/br.: pneumonitis/bronchiolitis (also see text).

## 3 Mechanisms of Drug Resistance

In this section, we analyze the mechanisms underlying both intrinsic and acquired drug resistance, with a particular focus on the critical role of TME in driving resistance development in certain cancer therapies. Additionally, drug resistance arising from epigenetic dysregulation and other often overlooked mechanisms is discussed. Antineoplastic agents rarely achieve definitive cures, even in highly chemo‐sensitive tumors. Drug resistance remains the primary cause of treatment failure and can arise from pre‐existing cellular characteristics (intrinsic resistance) or emerge as a consequence of therapy exposure (acquired resistance) [188]. A common clinical indicator of acquired resistance is the reduced duration of response to second‐line treatments compared with first‐line therapies [189]. Multiple genetic and nongenetic mechanisms contribute to drug resistance. Genetic factors include epigenetic modifications [190], enhanced DNA repair capacity [191, 192], gene amplifications [193], and the acquisition of drug‐resistant mutations.

Nongenetic mechanisms encompass the ability of a subset of tumor cells to survive chemotherapy by exploiting nutrients and signaling molecules within the TME [194, 195, 196].

### 3.1 Intrinsic Resistance

The most common contributors to intrinsic resistance include inherited DNA variants, subpopulations of resistant cancer cells, and the activity of specific intracellular pathways that counteract the effects of particular drugs. While some of these factors may be involved in both intrinsic and acquired resistance, inherited genetic variants uniquely characterize intrinsic resistance, as they are present prior to treatment initiation. Recent studies have reported various inherited genetic variants that affect drug response through different mechanisms [197].

For example, a study in NSCLC patients showed that the G allele of rs751402 in the ERCC5 promoter was associated with a poorer response to PBC compared with the A allele. This effect was likely due to increased ERCC5 expression and its role in nucleotide excision repair mechanisms [198]. Another variant in a regulatory region of ERCC5 (rs873601), which likely influences posttranscriptional regulation, was also linked to platinum resistance in lung cancer patients. Specifically, carriers of the GG genotype exhibited significantly shorter median OS than those carrying the A allele following PBC [199]. Further evidence for the role of inheritable variants in drug resistance was provided by Zhong et al. [200]. They found that the T allele of rs339331 within the RFX6 gene locus conferred intrinsic resistance to enzalutamide in prostate cancer.

Mechanistically, this allele creates an AR binding site within a regulatory region, enhancing RFX6 expression and resulting in increased resistance to enzalutamide [200]. Additional germline variants influencing drug response have been identified in CRC, such as rs199958833, which may affect 5‐FU sensitivity by destabilizing the cyclin‐dependent kinase 1 (CDK1) protein structure [201]. In breast cancer, numerous variants in genes encoding ATP synthase‐binding cassette (ABC) transporters, cytochrome P450 enzymes, glutathione S‐transferases, HER2, and others have been associated with therapy resistance [202].

Germline variants also impact the function of solute carriers (SLC), a protein family critical for drug uptake in cancer cells. Variants in SLC genes have been linked to drug resistance across various malignancies, including hematologic and solid tumors [203]. In contrast to germline variants, resistance associated with somatic mutations is more challenging to categorize as intrinsic or acquired. A recent large study in diffuse large B‐cell lymphoma highlighted the impact of pre‐existing mutations on drug resistance [204]. Specifically, mutations or copy number loss of the CD58 gene reduced the efficacy of chimeric antigen receptor T (CAR‐T) cell therapy. CD58 normally inhibits the JAK2/STAT1 pathway via LYN/CD22/SHP1, reducing PD‐L1 expression. Thus, CD58 inactivation or loss led to increased PD‐L1 levels, which impaired CAR‐T cell activity [204]. In NSCLC, an analysis of 578 patients with EGFR‐sensitizing mutations identified 16 individuals with intrinsic resistance to first‐line osimertinib treatment. Among these, 37.5% lost the EGFR‐sensitizing mutation, a phenomenon not observed in responders, suggesting a resistant subpopulation of cancer cells. These resistant patients also harbored off‐target mutations in TP53, RAS, PI3K, and RB1, potentially contributing to resistance via activation of oncogenic downstream pathways [205].

Intrinsic resistance can also result from activation of specific cellular pathways that diminish drug efficacy. For example, cancer cells can evade KRAS G12C inhibitors through activation of the YAP pathway or via epithelial‐to‐mesenchymal transition (EMT) processes [206]. Similarly, MAPK pathway activation confers intrinsic resistance to KRAS, MEK, and BRAF inhibitors [207] and has also been implicated in resistance to BET inhibitors in CRC [208].

### 3.2 Acquired Resistance

Acquired resistance to cancer therapies can arise through various mechanisms, including oncogene activation, mutations, or altered expression of the target gene, and activation of specific pathways induced by the therapy itself [209].

Acquired resistance has been documented across multiple therapeutic modalities and malignancies. TKI osimertinib is widely used in EGFR‐mutated NSCLC; however, resistance to this treatment almost inevitably develops. Two primary mechanisms have been described: EGFR‐independent (off‐target) and EGFR‐dependent (on‐target) resistance. Among off‐target mechanisms, MET gene amplification is the most common, though other alterations such as HER2 amplification, MYC, MDM2/CDK4, CCND1, PIK3CA, and KRAS mutations, BRAF fusions, and RB1 loss‐of‐function mutations have also been reported. These genetic changes activate downstream signaling pathways that bypass EGFR inhibition. On‐target resistance frequently involves mutations in EGFR itself, with C797S and T790M being the most prevalent. Less common EGFR mutations associated with resistance include L718Q, G796S, and S778I [210].

These findings were corroborated by Bayle et al. [211], who identified mutations in the EGFR pathway as well as alterations in PIK3CA, HER3, RET, BRAF, and NTRK1 as principal mechanisms of TKI resistance in NSCLC patients. Similarly, they reported that CRC patients developed resistance to anti‐EGFR monoclonal antibody therapies via mutations in EGFR, PIK3CA, HER2 amplification, MET amplification, and BRAF fusions [211]. Furthermore, the authors elucidated that acquired resistance mechanisms to antiandrogen therapy in prostate cancer involved the AR, including AR mutations, amplifications, and rearrangements [211].

Another mode of acquired resistance involves activation of intracellular signaling pathways. For instance, Egeland et al. [212] demonstrated that resistance to chemotherapy in a subset of triple‐negative breast cancer (TNBC) patients was driven by activation of SRC‐family kinases (SFKs) and MAPK/ERK signaling pathways.

SFKs have also been shown to be systematically activated following targeted inhibition of BRAFV600E‐mutant metastatic CRC, limiting the efficacy of this approach [213].

Moreover, KRAS‐ and BRAF‐mutant CRC can acquire resistance to anti‐EGFR therapies through activation of the PKC/CRAF/MEK/ERK signaling cascade, mediated by enhanced lipid metabolism. Wang et al. [214] reported an association between anti‐EGFR therapy resistance and intracellular accumulation of diacylglycerol (DAG), driven by increased activity of monoacylglycerol O‐acyltransferase 3. Mechanistically, elevated DAG promotes phosphorylation of PKC and CRAF, thereby activating MAPK signaling that counteracts the effects of BRAF and EGFR inhibitors [214].

Intracellular pathways can also be activated as an adaptive response that cancer cells employ to circumvent therapeutic effects. For example, activation of the RAS pathway through alternative upstream mediators such as EGFR, FGFR1, IGFR, and AXL has been observed following KRAS inhibitor therapy [206].

### 3.3 Tumor Microenvironment

The effectiveness of cancer therapies is profoundly influenced by the activity of immune, endothelial, and mesenchymal cells within the TME. Additionally, factors such as hypoxia, the composition of the extracellular matrix (ECM), and the secretion of specific soluble mediators by cells play crucial roles in this context. Consequently, the TME is a key determinant in the development of drug resistance for certain cancer treatments.

In breast cancer, TME‐associated resistance arises through multiple mechanisms, as comprehensively reviewed by Salemme et al. [215]. Type 2 tumor‐associated macrophages (TAMs) secrete various molecules into the TME, including IL‐2, IL‐6, IL‐10, basic fibroblast growth factor (FGF), tumor necrosis factor‐alpha, CCL2, and prostaglandin E2. These factors often activate signaling pathways such as PI3K/Akt/mTOR, EGFR, STAT3, and Bcl‐2, which mediate resistance to diverse chemotherapeutics [215]. Furthermore, TAMs suppress T cell effector function by releasing immunomodulatory factors including CCL20, CCL22, transforming growth factor beta (TGF‐β), IL‐6, and IL‐10, and by depleting l‐arginine levels within the TME through arginase 1‐mediated hydrolysis and nitric oxide synthase‐mediated metabolism [216].

Cancer‐associated fibroblasts (CAFs) also contribute to drug resistance by remodeling the ECM and limiting T cell infiltration into the TME. This remodeling activates the PI3K/AKT and Ras/Raf/MEK/ERK1/2 pathways, conferring resistance to multiple anticancer therapies [215], and increases ECM stiffness, fostering an immunosuppressive milieu [216]. Additionally, CAFs secrete soluble factors such as hepatocyte growth factor, which diminishes the efficacy of HER2‐targeted treatments, and pro‐inflammatory cytokines including TGF‐β, IL‐6, and IL‐8, which promote EMT. EMT enhances drug resistance by upregulating multidrug transporters and sustaining the cancer stem cell phenotype [216]. Secretion of IL‐6 and IL‐8 further contributes to immunotherapy resistance by facilitating the exclusion of CD8+ T cells from the TME [216]. CAFs also promote immunosuppression by expressing fibroblast activation protein, which amplifies TME inflammation via the STAT3–CCL2 axis, and by releasing CXCL12, which recruits CD4+ and CD25+ T cells and induces their differentiation into regulatory T cells (Tregs) [216].

Beyond TAM and CAF activity, the host immune response and immunotherapy efficacy are significantly compromised by hypoxia, a common feature in many tumors. In lung cancer mouse models, Robles‐Oteiza et al. [217] demonstrated that ICI‐resistant tumors exhibit elevated expression of hypoxia‐related genes, including those encoding glycolytic enzymes. Macrophages within the TME of resistant tumors also showed increased expression of glycolysis‐associated genes. Moreover, T cell infiltration was markedly reduced in hypoxic TME regions, even in tumors responsive to ICI, and hypoxia significantly suppressed major histocompatibility complex (MHC) class II expression on cancer cells [217]. Consistently, analysis of RNA sequencing data from lung cancer patients in the Stand Up To Cancer Mark Foundation dataset revealed that a “hypoxic metagene signature,” derived from ICI‐resistant cell lines, correlated with disease progression in patients [217].

### 3.4 Noncoding RNAs, Epigenetic Control, and Further Mechanisms

Another often overlooked mechanism contributing to drug resistance involves the dysregulation of noncoding RNAs and epigenetic processes, which can lead to aberrant activation or suppression of specific intracellular cancer‐related pathways.

In chemoresistant tumor cells, decreased levels of tumor‐suppressor microRNAs (miRNAs) alongside increased levels of oncogenic miRNAs promote multidrug resistance (MDR) [218]. In hepatocellular carcinoma (HCC), Borel et al. [219] identified 13 aberrantly expressed miRNAs associated with the overexpression of five ABC transporter genes, suggesting that miRNAs may drive hyperexpression of ABC transporters, particularly ABCB1, in HCC. Similarly, Li et al. [220] discovered 55 significantly dysregulated miRNAs in glioblastoma cells subjected to continuous temozolomide treatment via RNA sequencing. Concurrently, miRNA regulation of ABCG2/BCRP and ABCB1 was reported [221, 222]. Recent studies reveal that individual miRNAs can regulate multiple ABC transporters in chemoresistant tumor cells, potentially offering a more effective therapeutic strategy against MDR than the traditional one‐miRNA‐to‐one‐ABC‐transporter model. Long noncoding RNAs (lncRNAs) also influence ABC transporter‐mediated chemoresistance through interactions with miRNAs. Generally, lncRNAs are upregulated while miRNAs are downregulated, with miRNAs directly modulating ABC transporter expression. For example, in doxorubicin‐resistant osteosarcoma cells, elevated levels of lncRNA FOXC2–AS1 correlate with increased ABCB1 expression. Epigenetic regulation via lncRNA–protein interactions has been demonstrated in chemoresistant cancers, such as the overexpression of the methyltransferase EZH2 driven by lncRNA CASC9, which leads to upregulation of the MDR1 protein [223]. Circular RNAs (circRNAs) also play critical roles in various cancer processes, including chemoresistance [224]. One common mechanism is their function as competing endogenous RNAs, by sponging miRNAs. Studies show that circRNAs reduce intracellular drug accumulation by sequestering miRNAs, thereby indirectly promoting ABC transporter overexpression in chemoresistant cells. Additionally, tumor‐derived exosomes can transfer noncoding RNAs to tumor cells, modulating ABC transporter expression and contributing to cancer MDR [225].

Emerging evidence indicates that tumor cells employ multiple mechanisms to evade cytotoxic drug effects, predominantly through epigenetic reprogramming of gene expression. The two principal epigenetic modifications are: (1) DNA methylation and (2) histone modifications via acetylation or methylation. Both mechanisms differentially regulate chromatin accessibility, thereby influencing transcriptional activity [226]. It is estimated that over 30% of protein expression is controlled by endogenous miRNAs [227]. MiRNAs or their antagonists can modulate epigenetic regulators in cancer cells by targeting key proteins such as DNA methyltransferases, ten‐eleven translocation enzymes, and histone deacetylases (HDACs) [228]. DNA methylation, particularly in promoter regions, is commonly linked to gene silencing [229].

Alternative splicing, involving the removal of noncoding sequences from pre‐mRNA and rearrangement of coding segments, produces protein isoforms with diverse or even opposing functions. Dysregulation of alternative splicing contributes to numerous cancer hallmarks, including therapy resistance, by altering the expression of critical genes. Furthermore, a subpopulation of tumor cells can evade chemotherapy and targeted therapy by entering a reversible, slow‐proliferation state known as the drug‐tolerant persister (DTP) phenotype until additional resistance mechanisms emerge. Increasing evidence suggests that DTP cells adapt to varying microenvironments through modulation of epigenomic and transcriptomic profiles, metabolic reprogramming, and interactions with the TME [230].

## 4 Micrometastatic Disease: Biology and Challenges

Micrometastatic disease, occult DCCs, minimal residual disease (MiRD), and the more recently adopted term molecular residual disease are often used synonymously. Currently, residual micrometastatic disease following primary tumor resection, along with drug resistance, represent the major barriers to achieving a definitive cancer cure.

The mechanisms underlying the dissemination, dormancy, and reactivation of DCCs, as well as their detection and monitoring, have been extensively reviewed by us [231] and others [232, 233, 234, 235, 236, 237, 238]. More recently, these topics have also been comprehensively covered in two additional reviews, which readers may consult for further details [239, 240]. Here, we provide a brief summary and highlight the key features.

### 4.1 Biology of Micrometastases

Tumor dormancy is a pivotal concept in understanding residual micrometastatic disease, wherein cancer cells persist following primary treatment. This phenomenon encompasses two distinct, yet not mutually exclusive, forms: tumor mass dormancy and cellular dormancy. Tumor mass dormancy refers to a state of equilibrium within a tumor mass, characterized by a balance between cell proliferation and cell death, whereas cellular dormancy involves a slow‐cycling or nonproliferative state with reversible, transient growth arrest. Tumor mass dormancy is often termed “angiogenic dormancy” or “immunogenic dormancy,” reflecting the predominant regulatory mechanisms involved.

Angiogenic dormancy precedes the “angiogenic switch,” a transition marked by rapid tumor growth driven by neovascularization. The balance between proliferation and cell death critically depends on the availability of oxygen and nutrients supplied by pre‐existing blood vessels. Increased proliferation can deplete oxygen and nutrients within TME, especially in regions distant from vasculature, ultimately establishing an equilibrium between proliferation and cell death.

In immunogenic dormancy, this equilibrium is maintained through host immune surveillance, whereby T cells, B cells, and natural killer (NK) cells recognize and eliminate tumor cells via tumor‐specific or stress‐induced surface antigens. Additionally, interferon‐γ (IFN‐γ), produced by Th1 cells, CD8+ T cells, NK cells, and NKT cells, promotes dormancy through STAT1‐dependent and ‐independent pathways, including the indoleamine 2,3‐dioxygenase 1 (IDO1)–kynurenine (Kyn)–aryl hydrocarbon receptor (AhR)–p27 axis. Consequently, tumor cell growth is tightly regulated by the host immune system [239].

Cellular dormancy is characterized by quiescence, where solitary cancer cells undergo a reversible growth arrest, often linked to slow or halted progression through the G0‐to‐G1 phase of the cell cycle. DCCs originating from the primary tumor reach peripheral organs or tissues via the vasculature. These early‐seeder circulating tumor cells (CTCs) extravasate into the perivascular niche, where they receive physical support from vessel walls and mesenchymal stem cells (MSCs) acting as pericytes. The formation of the metastatic niche by early‐seeder CTCs is driven by tumor‐ and tissue‐specific gene expression patterns and chemokine secretion, a process termed “organotropism” or cancer cell “homing,” accounting for the divergent biology of metastatic niches compared with primary tumors [231].

Beyond reversible growth arrest, dormant cancer cells exhibit reduced metabolic activity and undergo chromatin and epigenetic alterations, facilitating durable dormancy. The niche is crucial for maintaining this state, involving pathways such as Wnt/Notch, TGFβ, mitogen‐ and stress‐activated kinase 1 (MSK1), Hedgehog proteins, and bone morphogenetic protein (BMP) signaling. These often converge on mTOR, MAPK/ERK, and p38 effector pathways, which are essential for niche maintenance. The small GTPase Cdc42 also contributes by enhancing p38 signaling and inducing growth arrest to sustain dormancy. At the metastatic niche, genetic and epigenetic mechanisms, alongside autophagy, angiogenesis, immune editing, hypoxia, and metabolism, cooperate with the TME to promote and sustain cancer dormancy and cell dormancy [240].

The awakening of dormant cancer cells is another critical aspect in residual micrometastatic disease biology. Breakdown of dormancy‐maintaining mechanisms occurs under the integrated influence of multiple factors, which can be categorized into nonimmunological and immunological mechanisms. Nonimmunological factors include systemic inflammation, ECM stiffness, aging TME, and physiological stresses that promote angiogenesis. ECM remodeling, interactions with stromal cells, nutrient availability, and other host factors also contribute. Furthermore, interactions with pericytes, fibroblasts, and vascular endothelial cells via growth factors, cytokines, and chemokines are pivotal in tumor relapse by reactivating slow‐cycling/dormant cancer cells [231, 239]. Immunological mechanisms involve bone marrow‐derived myeloid cells recruited to the metastatic niche, where they promote mesenchymal‐epithelial transition and growth of seeding EMT‐like cells. Myeloid‐derived suppressor cells (MDSCs) suppress immune responses in multiple organs. For example, MDSC‐secreted osteopontin inhibits CD8+ T cell proliferation in the lung metastatic niche. Tumor cell mTOR signaling recruits MDSCs via granulocyte‐colony stimulating factor (G‐CSF), which suppress T cell infiltration and induce DCC proliferation. CXCR2 signaling in neutrophils and MDSCs similarly inhibits CD3+ T cell‐mediated cancer cell killing in the liver metastatic niche. Post‐liver transplantation, elevated C‐X‐C motif chemokine ligand 10 and activation of toll‐like receptor 4‐expressing monocytic MDSCs contribute to HCC relapse. Macrophages in the mammary gland give rise to TAMs, promoting local and distant recurrence in TNBC.

Chronic inflammation also promotes awakening of dormant tumor cells. Cytotoxic proteins and proteases activate dormant cells in the lungs through integrin‐mediated focal adhesion kinase (FAK)/ERK/myosin light chain (MLC) kinase (MLCK)/Yes‐associated protein 1 signaling, driven by sequential protease‐mediated laminin remodeling. In the liver, dormant breast cancer cells are likely reactivated by liver‐specific pericytes secreting CXCL12, which induces CXCR4‐mediated NK cell quiescence and promotes CXCR4‐dependent outgrowth of dormant cancer cells [231, 239].

Last, the multifaceted roles of extracellular heat shock proteins (Hsp) in tumor dormancy and reactivation have been recently reviewed. The review describes in detail how these proteins, transported via vesicles and microvesicles, influence dormancy programs and tumor awakening [240]. In conclusion, dormant tumor cells within the metastatic niche can evolve to evade host immune surveillance, this favoring their eventual awakening and proliferation.

### 4.2 Detection and Monitoring

MiRD is commonly associated with involvement of the bone marrow or regional lymph nodes. According to the International Union Against Cancer, tumor cells measuring between 0.2 and 2 mm are classified as metastatic initiating cells [241]. Similarly, the sixth edition of the American Joint Committee on Cancer Staging Manual defines micrometastases as tumors smaller than 2 mm and macrometastases as those larger than 2 mm [242, 243]. DCCs from the primary tumor frequently spread to regional lymph nodes, bone marrow, and likely other sites. The latency period for these cells is thought to range from several months to many years, typically extending from before to after primary tumor detection [244, 245]. Clinically, sentinel lymph node (SLN) biopsy is the standard diagnostic technique in cutaneous malignant melanoma (CMM) and breast cancer to detect MiRD and improve primary tumor staging. Bone marrow biopsy is also performed in these and other cancers, with cytokeratins serving as conventional markers for identifying epithelial cancer cells in mesenchymal tissues [231]. Although the prognostic significance of MiRD detected in SLN biopsies in breast cancer remains controversial, a positive SLN biopsy in CMM significantly increases the risk of clinical tumor recurrence. Regarding MiRD detected via bone marrow biopsy, several clinical studies in solid tumors have identified it as an independent unfavorable prognostic factor by multivariate analysis [231]. For many years, serial serum tumor marker determination was the primary noninvasive method for postoperative monitoring to enable early detection of recurrence in patients who appeared disease‐free by conventional imaging. Breast, prostate, ovarian, and CRC were commonly monitored using serum tumor markers, with the LT—the interval by which marker elevation precedes instrumental detection of secondary lesions—well documented [246, 247, 248, 249, 250, 251, 252]. Extensive experience in dynamic evaluation of serial tumor markers we gained over time has primarily involved breast and CRC patients [246, 251]; consequently, this approach has been applied here to track MiRD progression and assess its kinetics in common solid tumors (see sub‐Section 6.1 and Supporting Information).

More recently, advances in technology have enabled detection of ctDNA and circulating cfDNA in blood samples, termed “liquid biopsy,” which has emerged as a novel, noninvasive tool for monitoring MiRD and predicting clinical recurrence. Several blood‐based monitoring studies have demonstrated that MiRD detection often correlates with clinical disease recurrence with an LT of a few months. However, it remains unclear whether treatment changes prompted by ctDNA detection improve clinical outcomes [253]. Consequently, numerous studies continue to evaluate the utility of liquid biopsy for detecting MiRD, clinical recurrence, and guiding adjuvant therapy. Concurrently, efforts to improve ctDNA detection sensitivity have focused on enhanced genomic profiling via ultra‐deep NGS. Broadly, two approaches are used: tumor‐informed and tumor‐agnostic assays. Tumor‐informed tests leverage prior genetic profiling of the primary tumor to improve MiRD detection sensitivity, while tumor‐agnostic assays perform variant calling without previous genetic information.

The personalized tumor‐informed approach targets pre‐existing mutations, enabling deeper sequencing. For example, in early‐stage NSCLC, the Patient‐specific pROgnostic and Potential tHErapeutic marker Tracking (PROPHET) assay utilized deep sequencing of 50 patient‐specific variants. PROPHET achieved 45% sensitivity at baseline and outperformed fixed‐panel assays prognostically, anticipating recurrence detected by radiology by a median of 299 days [254]. Another study, MRD‐EDGE, a machine learning (ML)‐guided whole genome sequencing platform for ctDNA single nucleotide variant and copy number variant detection, enhanced signal enrichment and improved MiRD detection across solid tumors. This tumor‐agnostic technology tracked changes in the low tumor burden setting following neoadjuvant immunotherapy in lung cancer [255].

In the cTRAK TN clinical trial [256], digital PCR (dPCR) and personalized multimutation sequencing—both tumor‐informed assays—were compared in 141 early‐stage TNBC patients. Personalized sequencing showed a median LT of 6.1 months from ctDNA detection to relapse versus 3.9 months for dPCR (*p* = 0.004, mixed‐effects Cox model). A further study [257] introduced new tracking technologies, including patient‐specific anchored‐multiplex PCR with cfDNA enrichment combined with an informatics tool (ECLIPSE) designed to detect low ctDNA levels (<1%), as commonly observed in early‐stage NSCLC.

Overall, these advanced technologies are currently not routinely implemented in clinical practice. Furthermore, these biomarkers cannot yet distinguish whether detected ctDNA originates from dormant versus proliferating tumor cells [258].

### 4.3 Therapeutic Vulnerabilities

Therapeutic vulnerabilities in MiRD that we have previously reported [231, 259] are briefly reviewed here.

DCCs likely acquire unique genetic features and gene expression signatures that contribute to heterogeneity among different metastases, as well as differences compared with the preinvasive or primary tumor lesions from which they originate [260, 261]. DCCs exit the proliferative cycle and enter a reversible G0–G1 cell cycle arrest, also referred to as a dormant or quiescent state [235, 236, 238]. Distinctions have been made between dormancy governed by tumor cell‐intrinsic pathways, termed “cell dormancy,” and dormancy regulated by the TME within the metastatic niche, known as “tumor dormancy.”

Accordingly, Phan and Croucher [262] described three modalities leading to dormant tumors: single early‐DCCs that survive at the periphery in a nonproliferative state (“cell dormancy”) and micrometastases derived from slow‐proliferating cells or cells in equilibrium between proliferation and death (“tumor dormancy”). Recent studies have identified several tumor‐intrinsic genes upregulated during dormancy, including BHLHE41, NR2F1, TGFB2, DAND5, HIST1H2BK, DDR1, IGFBP5, SOX9, HBP1, SNAI2, and CDKN1A. These genes contribute to the induction, maintenance, or prevention of awakening from dormancy. Other genes encode proteins that suppress metastasis; for example, KAI1 and KISS1 may promote cellular dormancy by inducing cell cycle exit, whereas MKK4, MKK7, NME1, and AKAP12 likely contribute to tumor dormancy by balancing proliferation and apoptosis [236]. Consequently, quiescent DCCs typically lack proliferation markers [263].

Moreover, CD4+ and CD8+ T cells play critical roles in inducing and maintaining dormancy in DCCs [236]. Specifically, CD8+ T cells induce quiescence via IFN‐γ production, which activates STAT1 signaling, thereby reducing proliferation and enhancing antitumor immunity [264]. In an immunogenic 3‐methylcholanthrene‐induced sarcoma model, when tumors became undetectable, the residual tumor burden was characterized by increased apoptosis and a low Ki67 proliferation index. Notably, tumor regrowth occurred following depletion of CD4+ and CD8+ T cells [265].

Additionally, several studies have reported that cancer cell proliferation and tumor growth are closely linked to immune inhibition and evasion mechanisms [266, 267]. Significant alterations in hematopoiesis have been observed in various human cancers and mouse models, including expansion of immature neutrophils and monocytes that migrate to the TME and contribute to immune suppression. The frequency of these bone marrow progenitors correlates positively with tumor burden [268]. The impact of tumor burden on the efficacy of ICIs has also been recently emphasized [269]. Consistent with this, clinical studies in advanced breast cancer and other malignancies demonstrate that an undetectable or nonproliferating tumor burden following standard or maintenance therapy is associated with improved prognosis. Some trials further indicate that this decrease in tumor burden correlates with enhanced immune responses, potentially augmented by concurrent immunomodulatory therapies [270].

Overall, quiescent metastatic niches and/or low tumor burden are likely associated with reduced immune suppression and greater potential for successful immune‐based therapeutic manipulation.

## 5 Emerging Strategies to Overcome Resistance and Eradicate Micrometastases

All patients with locally advanced disease or radically operable recurrence are potential candidates for alternative therapeutic strategies aimed at preventing the progression of MiRD to clinically overt metastatic disease [231, 259]. Recent advances in molecular biology have identified MiRD as a promising target for achieving a definitive cure in these high‐risk cancer patients.

In this section, currently proposed therapeutic strategies—based on experimental findings—are categorized into “next‐generation targeted agents,” “combinatorial approaches,” and “therapies targeting the TME.” For each category, strategies focusing on the “sleeping,” “killing or depletion,” and “awakening” of DCCs are actively being investigated [258, 271] in animal models and early‐phase I–II clinical trials [236, 239, 272].

Additionally, a novel immunotherapy regimen as well as emerging contributions from nanotechnology and AI‐driven predictive models are also discussed.

### 5.1 Next‐Generation Targeted Agents

#### 5.1.1 Sleeping Strategy

Hyperexpression of factors that promote dormancy or inhibit growth can induce or maintain the dormant state. Debio‐0719, a lysophosphatidic acid receptor 1 inhibitor, promotes breast cancer dormancy by decreasing proliferation markers Ki67 and ERK, while enhancing the p38 pathway, which suppresses growth [273].

Histone modifications and increased DNA methylation represent epigenetic mechanisms through which DCCs maintain dormancy. Accordingly, HDACs inhibitors among several epigenome‐targeting agents, have received US FDA approval and demonstrated efficacy in experimental studies. Specifically, HDACs inhibitors epigenetically upregulate the leukemia inhibitory factor receptor, thereby promoting dormancy in breast cancer cells [274, 275]. Similarly, they induce growth arrest and dormancy in uveal melanoma [276].

Other targets for inducing dormancy include MSK1, a positive regulator of metastatic dormancy in ER‐positive breast cancer, and regucalcin, which enhances key hallmarks of tumor dormancy in prostate cancer [277]. Ju et al. [278] identified COP9 signalosome 8 (CSN8) as essential for hypoxia‐induced dormancy in CRC; CSN8 overexpression activates hypoxia‐inducible factor 1α signaling, leading to upregulation of dormancy markers p27 and DEC2.

Preventing neovascularization is another established strategy to induce tumor dormancy, first demonstrated in 1972 by Gimbrone Jr. et al. [279], who observed that tumor fractions remained dormant in avascular microenvironments. Consistently, angiogenesis inhibitors such as angiostatin and agents targeting VEGF pathways promote dormancy by reducing blood supply and tumor growth [280, 281, 282, 283]. This strategy is further supported by the observation of significantly reduced vascular density in experimentally induced dormant tumors [258, 284].

#### 5.1.2 Direct Killing of Dormant Cancer Cells

Nimustine, an alkylating agent, completely eradicated tumors in a BRCA1/p53‐deficient mouse model, where platinum‐chemotherapy–refractory tumor cells remained sensitive to this drug [285]. Specifically, nimustine induces interstrand crosslinks that impair DDR in dormant cells. The regulator of G protein signaling 2 promotes dormancy and survival of dormant cells in NSCLC and its downregulation leads to apoptosis in these cells. Similarly treatment with phosphodiesterase 5 inhibitors has been shown to promote the proliferation of dormant SCCs, with enhanced antitumor efficacy when combined with chemotherapy [286].

The protein kinase RNA‐like endoplasmic reticulum kinase (PERK) also supports the survival of dormant cancer cells [287, 288]. Inhibition of PERK with the selective active inhibitor HC‐5404 eliminated dormant D‐Hep3 cells and dormant DCCs in the bone marrow [287]. This PERK inhibitor has demonstrated safety and efficacy in patients with advanced solid tumors and recently completed a phase I clinical trial [289]. Inhibition of the insulin‐like growth factor 1 receptor (IGF‐1R) reduced MiRD in an experimental pancreatic cancer model [290], while senolytic compounds have been shown to selectively eliminate dormant cancer cells exhibiting senescence‐like phenotypes [291].

Additional therapeutic approaches to directly eliminate disseminated dormant tumor cells include peptide‐mimetic copolymers and targeting the survival kinase dual‐specificity tyrosine phosphorylation‐regulated kinase 1B (DYRK1B). Peptide‐mimetic copolymers disrupt cell membranes to remove dormant PC‐3 prostate cancer cells resistant to docetaxel [292]. DYRK1B, which is overexpressed in dormant cancer cells, promotes dormancy by stabilizing CDK inhibitors and facilitating cyclin D degradation [293]. Knockdown or pharmacologic inhibition of DYRK1B induces apoptosis in various cell lines, contributing to the elimination of dormant cells [293].

### 5.2 Combinatorial Approaches

#### 5.2.1 Sleeping Strategy

In head and neck squamous cell carcinoma (HNSC), the retinoic acid–regulated orphan nuclear receptor NR2F1 promotes cellular dormancy [294]. C26, an NR2F1 agonist, induces dormancy in DCCs, thereby inhibiting lung metastasis [295]. Retinoic acid regulates NR2F1 expression [296], and all‐trans retinoic acid (atRA) has been shown to upregulate NR2F1 [294]. Conversely, DNA promoter methylation suppresses NR2F1 expression [294]. Accordingly, treatment with the DNA‐demethylating agent 5‐azacytidine combined with atRA induces NR2F1‐dependent dormancy [294]. A clinical trial investigating this combination in relapsed prostate cancer patients is currently ongoing (NCT03572387).

#### 5.2.2 Killing or Depletion Strategy

Inhibition of the SFK pathway induces dormancy in breast cancer cells, while combined inhibition of SFK and MEK1/2 eradicates dormant cells and reduces metastases [297]. Similarly, Src or COX‐2 inhibitors administered alongside chemotherapy suppress the proliferation of dormant SCC [298]. Several studies have shown that autophagy promotes and maintains dormancy in cancer cells, whereas mTOR complex 1, which regulates autophagy, supports their survival. Based on this, combining mTOR inhibitors with autophagy inhibitors has been proposed as a more effective therapeutic strategy [299, 300].

Hydroxychloroquine and other US FDA‐approved autophagy inhibitors have been tested alone or in combination with various agents to prevent relapse in breast cancer patients harboring bone marrow DCCs. In the CLEVER trial (NCT03032406), hydroxychloroquine combined with the mTOR inhibitor everolimus resulted in a reduction of DCCs alongside improved clinical outcomes [301, 302]. The ongoing ABBY (NCT04523857) and PALAVY (NCT04841148) trials aim to validate and extend these findings. In ABBY, hydroxychloroquine is administered with the CDK4/6 inhibitor abemaciclib, while PALAVY evaluates the safety and early efficacy of the checkpoint inhibitor avelumab or hydroxychloroquine, with or without the CDK4/6 inhibitor palbociclib. In both trials, bone marrow DCCs are the primary target, with eradication as the main objective.

Targeting Unc‐51 like autophagy activating kinase 1 in combination with CPT‐11 also reduces the proliferation of dormant cancer cells [303]. Finally, treatment with IFN‐γ in combination with inhibitors of IDO1 or the AhR suppresses dormant cancer cells [304, 305].

#### 5.2.3 Awakening Strategy

Combining a reactivation strategy with conventional chemotherapy, as suggested by many studies, could potentially exacerbate disease progression; therefore, careful management is essential. LB1, a protein phosphatase 2A inhibitor, can reactivate dormant cancer cells by activating AKT and interfering with p53‐mediated cell cycle arrest, thereby sensitizing cells to cytotoxic agents. This mechanism has been validated in the treatment of HNSCC [306], glioblastoma multiforme, and neuroblastoma [307]. DTX‐P7 is a peptide–drug conjugate that combines the heptapeptide P7 with docetaxel. The P7 peptide specifically binds to the Hsp90 on the surface of NSCLC cells. This conjugate induces dormant A549/CD133+ cells to re‐enter the cell cycle by degrading a negative regulator of cell cycle progression, followed by targeted killing through docetaxel release [308]. Among other potential targets is the loss of TGF‐β‐SMAD4 signaling, which can trigger the activation of dormant DCCs [309]; thus, targeting SMAD4 may actually promote cell cycle re‐entry. Fbxw7 is highly expressed in dormant breast cancer cells; its knockdown reactivates dormant cells, and combining Fbxw7 knockdown with cytotoxic therapy reduces DCC burden [310].

Disseminated dormant breast cancer cells typically exhibit a mesenchymal phenotype [311], and their transition back to an epithelial state via E‐cadherin upregulation can induce reawakening [312]. Therefore, modulating E‐cadherin expression may influence the dormancy‐to‐proliferation switch, potentially driving disseminated dormant breast cancer cells to re‐enter the cell cycle.

### 5.3 Targeting the TME

#### 5.3.1 Sleeping Strategy

Metastatic and premetastatic niches are specialized microenvironments that undergo continuous remodeling driven by ECM modifications and tumor‐specific signals. The interactions between DCCs and the ECM play a critical role and represent potential therapeutic targets. Often, the TME within the metastatic niche induces cell dormancy, particularly through signals that inhibit proliferation while promoting survival. Notably, microenvironmental factors such as TGFβ2 [313, 314, 315, 316], BMP7 [315, 317, 318], and IFN‐γ [319, 320, 321] promote dormancy in DCCs. Consequently, treatment with soluble TGFβ2 or BMP7 constitutes a dormancy‐inducing (or “sleeping”) strategy.

Inhibiting growth‐promoting factors—including urokinase receptors [322, 323], ERK signaling [297], and cell cycle kinases [287, 294]—can also enforce dormancy in cancer cells. Specifically, ECM components such as type I collagen and fibronectin drive the transition of dormant to proliferative breast cancer cells. This process is mediated by integrin β1 activation, which triggers phosphorylation of ERK‐dependent MLC via MLCK [324, 325]. The observation that integrin β1‐deficient tumors exhibit prolonged dormancy further underscores the critical role of integrin β1 in promoting tumor cell proliferation [326]. Accordingly, inhibition of integrin‐mediated signaling pathways has been proposed as a dormancy‐inducing strategy [324, 325, 327].

Osteoblast‐induced dormancy has been demonstrated in prostate cancer cells, with the FAK inhibitor PF‐562271 recapitulating osteoblast‐mediated dormancy [328]. Hypoxia is another TME factor known to induce dormancy across various tumor types [278, 329, 330, 331, 332]. In breast cancer, cells remodel fibronectin to maintain dormancy through α5β1 integrin‐mediated adhesion and Rho‐associated kinase‐mediated cellular tension [314]. Furthermore, ER‐positive breast cancer cells, via fibronectin and resident FGF‐2, increase the population of dormant cells [333]. Similarly, ECM components can promote DCC dormancy through interactions with syndecan receptors [334].

#### 5.3.2 Killing or Depletion Strategy

In pancreatic cancer models, linsitinib, which inhibits IGF1 or IGF‐1 receptor along with AKT, favors the elimination of dormant cancer cells not expressing mutant KRAS or c‐MYC [290].

#### 5.3.3 Awakening Strategy

Cells surrounding micrometastases regulate dormancy or proliferation through direct interactions and the release of cytokines and environmental cues [300]. An osteoclastic niche, promoted by vascular cell adhesion molecule 1, facilitates dormancy escape of breast cancer cells and activates micrometastases [335]. Within the micrometastatic bone marrow niche, NG2+/Nestin+ MSCs induce dormancy of disseminated breast cancer cells via TGFβ2 and BMP7; conversely, MSC depletion or TGFβ2 knockout in MSCs reactivates dormant cells [315]. The level of TGFβ2/MAPK signaling is critical, and TGFβ exhibits organ‐specific roles in dormancy regulation. For example, the secreted TGF‐β ligand Coco promotes reawakening of dormant breast cancer cells in the lung by antagonizing BMP signaling, a mechanism not observed in bone or brain tissues [336]. In breast cancer models, delivery of IL‐6 and G‐CSF activates the MEK/ERK pathway, triggering awakening of dormant cells [337]. Both IGF1 and G‐CSF have been implicated in influencing dormant cell behavior; specifically, IGF1 administration promotes proliferation of dormant breast cancer cells, as evidenced by increased Ki67+ DCCs [338]. The main therapeutic strategies targeting MiRD are summarized in Table 7.

**TABLE 7:** Main therapeutic strategies targeting micrometastases.

Therapeutic strategy	Mechanism	Treatment/compound	References
Sleeping	Inhibition of LPA‐1	DEBIO‐0719	[273]
	Epigenetic	HDACis	[274, 275, 276]
	Induction of NR2F1 or NR2F1 agonist	AZA plus atRA or C26	[294, 295]
	TME factors inducing dormancy	TGF‐beta2 or BMP7 soluble factors	[313, 317]
	FAK inhibition	PF‐562271	[322, 328]
	Integrin‐mediated signaling pathways	Inhibitors	[323, 324, 325]
Killing/depletion	Impaired DNA damage repair	Nimustine	[285]
	RGS2 promoted dormancy	RGS2 genomic ablation or PDE5 inhibitors	[286]
	PERK inducing survival	HC54‐04 selective PERK inhibitor	[289]
	IGF1/Akt signaling inhibition	Limsitinib (OSI‐906)	[290]
	Dormant DCCs with senescence‐like phenotypes	Senolytic drugs	[291]
	SFK signaling	Ct combined with Src or COX2 inhibitors	[298]
	Autophagy regulation	Hydroxychloroquine alone or with mTOR inhibitors	[302, 303]
	INF‐gamma through IDO/Kyn/AHR/p27 cascade	IDO and integrin alpha2beta1 signal pathway inhibitors	[305]
Awakening	PP2A inhibition	LB1	[306, 307]
	Negative regulator of cell cycle progression plus Ct	DTX‐P7	[308]

Abbreviations: LPA‐1: lysophosphatidic acid receptor 1; NR2F1: orphan nuclear receptor; AZA: demethylating agent 5‐azacytidine; atRA: all‐transretinoic acid; TGF: tumor growth factor; RGS: regulator of G protein signalling; SFK: src family kinase; Ct: chemotherapy; COX2: cyclooxygenase‐2; INF: interferon; IDO: indolamine 2,3 dioxygenase 1; AHR: aryl hydrocarbon receptor; IGF: insulin‐growth factor; Akt: protein kinase B; PP2A: protein phosphatase 2A; DTX‐P7: heptapeptide P7 combined with docetaxel; PDE: phosphodiesterase (also see text).

#### 5.3.4 Immunotherapy

Immune cells are a key component of the TME, and immune‐related mechanisms can prevent disease progression by maintaining tumor cell dormancy. Tumors derived from the 4T07 mouse breast cancer cell line exhibit a higher frequency of CD39+PD1+CD8+ T cells compared with those from the 4T1 line. The 4T07 tumors prime CD8+ T cells to promote dormancy and prevent relapse [321]. Intratumoral immunotherapy using a toll‐like receptor 9 ligand combined with an anti‐OX40 antibody controlled tumor growth in 4T1 breast cancer, CT26 colon cancer, and B16–F10 melanoma models. Furthermore, this dual immunotherapy reduced tumor burden in mammary glands and inhibited lung metastases in the MMTV–PyMT spontaneous breast cancer mouse model. The antitumor response and metastasis prevention were likely mediated by increased activity of CD4+ and CD8+ Th1 immune cells [339].

Conversely, dormant cancer cells can evade immune surveillance by downregulating NK cell activity, thereby escaping NK cell recognition and cytotoxicity, while maintaining a stem‐like state conducive to dissemination [321]. Dormant cells may also reduce MHC‐I expression to avoid detection by CD8+ T cells [340, 341]. Enhancing antigen‐specific T cells capable of recognizing and eliminating dormant cells represents another strategy to counter immune evasion. This can be achieved through T cell‐based vaccines, adoptive transfer of engineered T cell receptors, and CAR‐T cell therapies [342]. Moreover, dormant tumor cells promote an immunosuppressive TME characterized by reduced TILs and dysfunctional T cells [343]. PD‐1 and PD‐L1 are immune checkpoint receptors on T and B cells that facilitate immune escape by downregulating antitumor responses. Anti‐PD‐1 agents reduce dormant DCCs in the lungs of mice [321], suggesting that PD‐1/PD‐L1 inhibitors may be effective in eliminating dormant cancer cells and preventing tumor relapse [344, 345]. This therapeutic approach is currently under evaluation in a phase II clinical trial recruiting breast cancer patients with bone marrow DCCs (NCT04841148). Similarly, in the 4T1 TNBC mouse model, combined anti‐PD‐L1 and anti‐CD47 antibody treatment inhibited tumor proliferation and CTC–mediated lung metastasis [346].

Building on the above mentioned MiRD therapeutic vulnerabilities, we recently proposed a drug regimen aimed at inhibiting immune suppression, termed IS‐IIT. We have reported preliminary evidence supporting its efficacy in a small cohort, including two patients with biochemical recurrence (BCR) following radical prostatectomy and two additional cancer patients with likely MiRD after primary tumor removal [347]. DCCs exhibit genetic features and gene signatures, loss of proliferation markers, and partial acquisition of stem‐like properties that confer intrinsic resistance to conventional therapies [260, 269] targeting actively dividing cells [263], as well as to targeted anticancer agents [236]. However, the reduced metabolic activity of quiescent DCCs helps prevent cellular exhaustion and decreases the likelihood of acquired resistance via oncogenic mutations during cell division [249]. Our immune modulation approach aims to inhibit immune suppression mediated by MDSCs, Tregs and macrophages [347], thereby moving the balance of physiological immune surveillance toward an effective anticancer immune response and “immunogenic dormancy.” This strategy is anticipated to be less susceptible to drug resistance and effective across different tissues, irrespective of DCC phenotype [231]. Here, we provide an update on two previously reported prostate cancer cases with BCR and two similar newly recruited patients currently receiving cycles of IS‐IIT. As summarized in Table 8, these four prostate cancer patients were followed for 42 to 134 months post‐BCR. Each patient underwent 29 to 70 IS‐IIT cycles aimed at counteracting BCR. Patient 2, after local recurrence characterized by a 27x26 mm nodule, received salvage radiotherapy and subsequently commenced IS‐IIT alone during BCR without ADT. The other three patients received IS‐IIT cycles alongside three to six injections of triptorelin 11.25 mg. In all patients, IS‐IIT was initiated immediately upon serum total PSA rising above 0.2 ng/mL on two consecutive measurements, indicative of BCR. When PSA approached 1.5 ng/mL, patients received the first triptorelin injection, during which IS‐IIT was temporarily paused and resumed 1 month later. This cycle continued with IS‐IIT administered until PSA again approached 1.5 ng/mL. The 1.5 ng/mL threshold for triptorelin administration was arbitrarily set to maintain a low micrometastatic tumor burden.

**TABLE 8:** Main findings in four prostate cancer patients who after radical prostatectomy (RP) with (*N* = 1) or without (*N* = 3) radiotherapy showed BCR treated with a novel schedule of IS‐IIT alternated with ADT; so far, none of them has progressed to clinically overt metastatic disease.

Patient							Last PSA					
*N*	Age (yrs)	RP (date)	Gleason (grade)	pTNM	DFI^a^ (mo)	PSA at BCR^b^ (ng/mL)	ng/mL	date	Follow‐up from BCR (mo)	IS‐IIT cycles (N)	TPTR injections (N)	Adjuvant RT
(1) RG	65	12‐01‐2010	4 + 4 (4)	pT3bN0M0	36	0.23	0.05	11‐03‐25	143	82	7	No
(2) BS	57	09‐10‐2009	4 + 5 (1)	pT3aN0Mx	133^c^	0.24 ^c1^	<0.01	10‐12‐24	127^c2^	85	No	No
(3) DE	71	11‐02‐2019	4 + 4 (4)	pT3bN0R1M0	3	0.67	<0.35	24‐04‐25	72	37	4	Yes
(4) LG	75	12‐06‐2020	3 + 4 (2)	pT3bN0R1Mx	5	0.23	0.97	14‐03‐25	51	27	3	No

^a^ Time from RP to BCR.

^b^ Last value after BCR and before the beginning of treatment.

^c^ Time to local recurrence (27x26 mm nodule) treated with salvage radiotherapy (RT).

^c1^ Value before the beginning of IS‐IIT, 24 months after salvage RT.

^c2^ Time following salvage RT; TPTR: triptorelin; also see reference [347] and text.

These findings suggest that IS‐IIT cycles, initiated soon after BCR, substantially slowed PSA progression and synergized with triptorelin to further reduce PSA levels. To date, in these high‐risk patients, prolonged IS‐IIT combined with intermittent triptorelin injections has prevented BCR progression to clinically overt metastatic disease (Table 8).

### 5.4 Nanotechnology and Drug Delivery

The field of nanotechnology for drug delivery dates back to the 1960s, when liposome structures were first characterized. However, it took nearly 30 years before the first nanomedicines entered clinical use. Here, we briefly examine the current role of nanotechnology in targeting MiRD.

The evolution of nanomedicines as nanoparticles (NPs), also known as nano‐carriers (NCs), with diameters ranging from 1 to 100 nm, reflects advances in nanomedical engineering and nanomedicine. Polymeric, inorganic, and lipid‐based NCs are the most common forms, each tailored to meet specific therapeutic needs. NCs serve as delivery vehicles that transport active substances to their intended targets, which may be tissues, cells, or even individual molecules [348]. These active substances can be conventionally synthesized molecules, nanotechnology‐enhanced agents, or entirely novel nanotechnology‐based compounds.

The clinical approval of NP‐based agents faces multiple challenges [349]. When applied clinically, they often exhibit limited specificity, reduced efficacy, and require higher doses, which can increase toxicity and necessitate adjunctive medications with their own side effects. This, in turn, may further compromise patient health and increase treatment costs [350, 351]. Consistent with the “magic bullet” therapeutic concept [352], the ideal drug selectively targets diseased cells with minimal or no off‐target effects. To achieve this goal, nanotechnological approaches for targeted drug delivery have expanded significantly over the past two decades, fueled by ongoing advances in basic research and the development of nanomaterials with improved properties [353, 354].

Despite these advances, relatively few NP‐based therapeutics have received regulatory approval and entered clinical practice, and none specifically target micrometastases. A recent study utilizing imaging methods to quantify NP delivery to micrometastases concluded that developing predictive models based on micrometastasis physiology could enable personalized treatments tailored to individual patient microenvironments [355].

In EOC, micrometastases persist within the abdominal cavity, contributing to tumor recurrence and rendering the disease incurable. A recent review [356] examined therapeutic NC delivery strategies spanning various mechanisms of action. For example, small interfering RNA therapies designed to resensitize tumor cells to chemotherapeutics were distinguished from those aimed at reducing proliferation and tumor recurrence. Despite promising preclinical results, no such strategies have yet reached clinical application. Alpha‐particle emitters exhibit potent antitumor activity, primarily through elimination of MiRD. One study [357] encapsulated alpha emitters such as ^225^Ac within polymeric NCs (polymersomes) to reduce systemic toxicity. These polymersomes penetrated throughout U87 human glioma spheroids, delivering radiation uniformly and significantly inhibiting spheroid proliferation at low doses (0.1 kBq ^225^Ac).

Finally, miRNAs represent promising therapeutic targets, and developing efficient miRNA delivery systems is a key objective. Nanomaterials enhance miRNA stability in circulation, improve cellular uptake, and enable targeted delivery to specific organs or cell types. A recent review [358] highlights the critical role of nanotechnology in advancing miRNA‐based therapeutics, emphasizing its potential to accelerate progress in this emerging field.

### 5.5 AI‐Driven Predictive Models

AI algorithms can effectively capture tumor complexity arising from the interplay between genes and their downstream effects within biological network structures [359, 360], enabling the exploration of novel anticancer targets [361, 362]. A recent review examined the rationale and principles behind commonly used AI algorithms and highlighted their applications in tumor target detection and drug discovery [363].

With advances in high‐throughput sequencing, the field of omics has rapidly expanded, offering unprecedented opportunities alongside significant challenges in data analysis and interpretation for predictive modeling in precision medicine. Deep learning (DL), particularly convolutional neural networks, has driven impressive progress in predictive modeling. This approach not only improves predictive accuracy but also employs transfer learning to optimize computational efficiency and model performance. However, challenges remain, including model interpretability, data volume and heterogeneity, and the limited ability of current models to fully capture intrinsic biological complexity. Large datasets provide valuable insights into molecular processes and opportunities for modeling drug efficacy prediction. Yet, the increasing complexity of models may reduce their utility for biological discovery and undermine confidence in clinical application. AI and ML‐based methodologies [364, 365] offer novel strategies to overcome these limitations by enhancing knowledge integration across multiple levels of biological organization. ML algorithms encompass a diverse array of computational techniques that facilitate the development of predictive models yielding actionable biological insights. These methods have demonstrated efficacy in processing high‐dimensional datasets and have proven valuable in various genomic research contexts. However, as data volume and dimensionality increase, the limitations of classical methods become more pronounced. The emergence of DL has initiated a paradigm shift in AI, fundamentally transforming data analytics. DL's capacity for automated hierarchical feature learning from raw data enables to be of significant value in predictive modeling and to capture complex interdependencies within noisy, high‐dimensional datasets effectively.

Transfer learning further facilitates the repurposing of knowledge gained from large datasets to improve accuracy in smaller cohorts [366]. Reflecting recent advances in multiomics integration, a recent scholarly review proposed a novel AI‐driven, biology‐inspired multiscale modeling framework designed to synthesize multiomics data across biological levels, organismal hierarchies, and species boundaries. This framework aims to predict genotype–environment–phenotype interactions under diverse conditions [367]. The authors suggest that “AI models inspired by biology may identify novel molecular targets, biomarkers, pharmaceutical agents, and personalized medicines for presently unmet medical needs.” Additionally, a separate review detailed advances in ligand‐binding site prediction through geometric DL and sequence‐based embeddings, facilitating the identification of putative druggable target sites [368]. Looking ahead, adaptive clinical trial platforms—such as basket trials (targeting different cancers with a shared mutation or biomarker) and umbrella trials (targeting a single cancer type with different genetic alterations receiving tailored therapies)—are expected to become more widespread. These developments will depend on biomarker‐driven patient stratification and improved understanding of ctDNA and cfDNA roles in detecting MiRD, clinical recurrence, and guiding adjuvant therapy.

In conclusion, AI‐driven predictive models have the potential to address key challenges in micrometastatic disease, including the identification of reliable biomarkers for improved detection, imaging, and postoperative monitoring, as well as the discovery of suitable therapeutic targets and drug candidates.

## 6 The Kinetics of DCCs Growth and a Protocol to Counteract Micrometastatic Progression in Solid Tumors at High Risk of Relapse

This section deals with the kinetics of DCCs growth and the mechanistic rationale of a proposed protocol to counteract the progression of DCCs.

### 6.1 The Kinetics of DCCs Growth

A micrometastasis becomes detectable when it reaches a volume threshold of approximately 0.5 cm for positron emission tomography/computed tomography (CT) imaging, which corresponds to the accuracy of a single voxel, or roughly 0.065 cm^3^ [244, 369]. It is estimated that an average 1 cm^3^ tumor nodule contains approximately 10⁹ cells and weighs about 1 g [370]. The growth dynamics of DCCs—from the onset of irreversible proliferation to eventual radiographic detection—remain poorly understood, as the tumor burden typically remains undetectable for most of this time. Growth is commonly inferred through mathematical models based on the longitudinal measurement of secondary lesions after they become visible.

Three widely used models to approximate the growth kinetics of primary tumors and micrometastases are exponential, logistic, and Gompertzian [234, 244, 371]. Of these, the Gompertzian model generally provides a better fit for primary tumor growth, whereas metastatic progression often follows an exponential trajectory, particularly during the clinically observable period [234, 371]. It has been noted that “a cell destined to become part of an oligo‐metastatic distribution must undergo about 30 doublings to become clinically detectable as an overt metastasis (2^3^⁰ or 10⁹ cells),” and that “the actual time to appearance of a solitary metastasis, or of oligo‐metastases, in any particular patient will depend on the growth rate of the metastases in that individual, but will always require about 30 volume doublings” [371]. As previously mentioned, we used serum tumor markers and their LT to track and assess the kinetics of MiRD progression.

In breast cancer patients, we conducted a study involving intensive postoperative surveillance using more sensitive thresholds called “individual reference limits” (IRLs) for the serum tumor markers CEA, TPA, and CA 15.3. The mean LTs observed were 7, 8.8, and 8.5 months, respectively [246]. Similar longitudinal studies have been conducted by others: in ovarian cancer using CA 125 [247, 248] or HE4 [249, 372]; in CRC using CEA [252, 373, 374, 375]; and in prostate cancer using PSA, where a temporal correlation between BCR and bone metastases was observed [376]. The mean LTs found in these studies for CEA, TPA, CA 15.3, PSA, CA 125, and HE4 are summarized in Table 9. Notably, the median LT across these four cancer types was approximately 7 months, overall to be considered the median LT of a tumor with intermediate level of biological aggressiveness.

**TABLE 9:** Overall median lead time (mo.) obtained from mean serum CEA, TPA, CA15.3, CA125, HE4, and PSA lead time for “early” detection of relapse during postoperative monitoring of untreated, disease‐free breast, colorectal, ovarian, and prostate cancer patients.

Cancer type	Mean serum tumor marker lead time (mo.)						Overall median lead time (mo.)
	CEA	TPA	CA15.3	CA125	HE4	PSA	
Breast	7	8.8	8.5	—	—	—	7
Colorectal	5.9; 10.3; 10	—	—	—	—	—	
Ovarian	—	—	—	4; 5.4 (6, 9, 6, 4, 7, 2, 4)^a^	2 (1–3)^b^ 6.5 (5–8)^b^	—	
Prostate	—	—	—	—	—	10 (8, 12)^a^	

^a^ Single observation.

^b^ Ranging value; also see Refs. [246, 247, 248, 249, 252, 372, 373, 374, 375, 376] and text.

To better characterize the growth dynamics of DCCs and optimize the duration of IS‐IIT cycles, we calculated the mean serum tumor marker LT using experimental data from 10 untreated or drug‐resistant patients with the same tumor types enrolled at our oncologic center: prostate (*n* = 2), ovarian (*n* = 1), colorectal (*n* = 1), and breast (*n* = 6). The ovarian cancer patient experienced two separate recurrences, bringing the total number of recurrence events to 11. For each recurrence, a growth curve was constructed based on longitudinal serum tumor marker values. An exponential model was then applied to estimate the tumor growth rate. These real‐world clinical data more accurately reflected the extended time course of micrometastatic growth, even while still below the threshold of imaging detection. The calculated median serum LT was 9.3 months—approximately 2 months longer than the median derived from previously published data (see Table 9).

Importantly, in six of the 11 recurrences (54%), the IRL (in five cases) and BCR (in one case) were the relevant thresholds, rather than the conventional laboratory reference values typically used in the literature. These thresholds proved to be more sensitive for early detection. Kinetic analysis also enabled us to estimate the mean tumor doubling time (TDT), which was 136 days. This value is comparable to the TDT reported for colorectal lung metastases, which ranges from 109 to 171 days [234, 377, 378], and to a separate study reporting a mean TDT of approximately 130 days for secondary pulmonary nodules of varying sizes and tumor origins [379]. Notably, in those studies, TDT was calculated using serial roentgenographic or CT imaging, while our approach was based on tumor marker kinetics. Table S1, in Supporting Information, reports some parameters estimated by our mathematical model including the TDT, the cut‐off time and the LT, deduced uniquely by the serological tests performed on several patients affected by different kind of cancer. The mean and the median of these values are reported in Table S2. Figures S1 and S2 in Supporting Information show the approximation of the serological values together with the extrapolation performed by means of a “log least‐square” technique.

### 6.2 The Mechanistic Rationale

We have recently characterized the dormant state of DCCs as an unstable virtual equilibrium (defined as a “virtual equilibrium line = 0”), which can be disrupted at any time by cellular awakening and proliferation. It is hypothesized that dormant DCCs in secondary tissues may evade conventional anticancer immune responses through a process of immune editing [236]. By exploiting the transiently immunosuppressive TME, these cells can reactivate and progress into clinically overt metastatic tumors.

**FIGURE 1:**
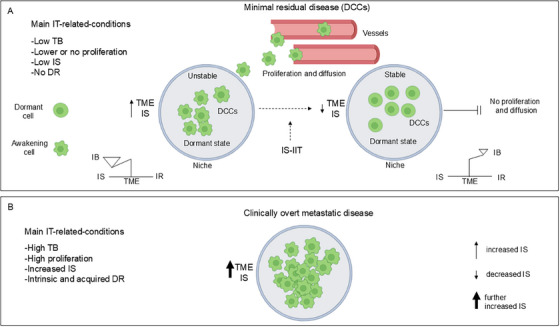
In TME, undetectable DCCs (panel A) join with a low tumor burden along with low immune suppression, that favor successful immune manipulation through immune suppression inhibiting immune‐therapy. This moves the immune balance towards the immune response and likely makes more stable the dormant state of DCCs in the unstable metastatic niche and/or allows innate and adaptive immune response to kill the cancer cells. In clinically overt metastatic disease (panel B), higher tumor burden and immune suppression favor cancer cell proliferation and the arising of drug resistance. DCCs, disseminated cancer cells; DR, drug resistance; IB, immune balance; IR, immune response (NK, CD4+ and CD8+ T cells); IS‐IIT, immune suppression inhibiting immunotherapy; IS, immune suppression (Tregs, macrophages, MDSCs); IT, immune‐therapy; TB, tumor burden; TME, tumor microenvironment.

We propose that IS‐IIT cycles may shift this dynamic balance toward enhanced immune response thereby stabilizing the unstable dormant state and/or directly eliminating the DCCs (Figure 1).

Based on our kinetic analysis of DCC growth, we estimated a median LT of approximately 9 months for tumors with intermediate biological aggressiveness. For tumors with low or high aggressiveness, this value may vary by approximately ±2 months. Considering the possibility of proliferating foci escaping IS‐IIT, we subtracted an additional 2 months from each interval to allow conventional therapies to intercept progressing MiRD. The recommended intervals for alternating IS‐IIT and conventional antiproliferative therapies in tumors of varying biological aggressiveness are outlined in Table 10. For patients with nonendocrine‐dependent cancers and intermediate biological aggressiveness, following completion of standard adjuvant chemotherapy, our proposed protocol includes cyclic IS‐IIT for 7 months, followed by four cycles of immune‐modulating conventional chemotherapy. These cycles should be alternated continuously over a 5‐year period.

**TABLE 10:** Additional adjuvant therapy proposed in solid tumors in patients with locally advanced disease or radically resected metastases.

	Immunotherapy (IT)^a^				Conventional therapy^a^		
	IS‐IIT				Years 1–7		
	Years 1–5		Years 6–7		Hormone therapy^c^	Chemotherapy	
Tumor biological aggressiveness^b^	Interval time (mo.)	Cycles (*N*)	Interval time (mo.)	Cycles (*N*)	Interval time^d^ (mo.)	Cycles (*N*)	Interval time (mo.)
Low	9	6–7	11	8	5–6	4	3–4
Intermediate	7	5	9	6–7			
High	5	3–4	7	5			

^a^ Conventional therapy (hormone therapy or chemotherapy) follows IT; they both are alternatively and continuously given up to 7 years.

^b^ In locally advanced disease, assessment is based on type, grade, number of involved loco‐regional lymph nodes and proliferation index (when available); in recurred patients, assessment is based on the type of primary tumor, disease‐free interval, number, and site of radically resected metastases.

^c^ It refers to breast cancer only.

^d^ For prostate cancer, also see Ref. [347] and text.

For tumors with high or low biological aggressiveness, the IS‐IIT cycle duration should be adjusted to 5 or 9 months, respectively, as described above. Biological aggressiveness should be assessed based on histology, tumor grade, the number of involved loco‐regional lymph nodes, and the proliferation index. In cases of relapsed disease following radical resection, at least an intermediate level of aggressiveness should be assumed. Factors such as primary tumor type, disease‐free interval, number and site of radically resected metastases, and additional prognostic biomarkers may assist in identifying patients with high‐risk biology.

For endocrine‐dependent cancers—particularly prostate cancer—our recent findings (see Table 8) suggest revising previous recommendations. Specifically, we propose confirming the protocol in patients with BCR using a serum PSA threshold of 1.5 ng/mL as the trigger to initiate ADT [231]. In endocrine‐dependent breast cancer, IS‐IIT cycles should be alternated with selective estrogen receptor modulators/selective estrogen downregulators or aromatase inhibitors for 5–6 months, continuing for up to 7 years. IS‐IIT has been well tolerated with minimal side effects [347]. The relatively short treatment intervals proposed for conventional hormone or chemotherapy are expected to significantly reduce the risk of acquired drug resistance. If no clinically overt metastatic disease develops within the first 5 years, the IS‐IIT cycle interval should be extended by 2 months during years 6 and 7. However, the interval for conventional chemo‐ or hormone therapy should remain unchanged.

During this extended treatment phase, the patient's immune system—repeatedly challenged by multiple episodes of proliferative activity—is more likely to acquire the capability through the memory T cells of effectively controlling future micrometastatic progressions. The proposed schedule for extended adjuvant immune, hormonal, or chemotherapeutic treatment in high‐risk cancer patients is provided in Table 10.

## 7 Conclusions and Prospects

High‐risk patients with solid tumors frequently develop clinically overt metastatic disease, which remains the leading cause of mortality in this population. The era of targeted therapies, which began over two decades ago with high expectations, has undoubtedly led to significant advances in molecular biology and genetics, fundamentally transforming cancer treatment paradigms. While some patient subsets have experienced substantial improvements in disease‐free survival and/or OS, the majority of patients have derived only marginal benefits. More importantly, even among initially responsive patients, the emergence of intrinsic or acquired drug resistance renders metastatic disease largely incurable. Despite these limitations, multinational pharmaceutical companies continue to heavily invest in the targeted therapy paradigm, often in collaboration with institutional researchers and public funding frequently at the expense of exploring promising alternative strategies.

This review begins by summarizing key findings from major clinical trials involving targeted therapies in the most common advanced solid tumors, along with an analysis of the primary mechanisms underlying drug resistance. It then explores how recent advances in our understanding of micrometastatic seeding and latency in peripheral tissues account for development of novel therapeutic strategies aimed at preventing micrometastatic disease progression. In the subsequent sections, we discuss the potential of nanotechnology and AI‐driven predictive modeling to address and overcome therapeutic challenges in this evolving field.

Furthermore, insights into MiRD and the immunological conditions favoring its manipulation have allowed us to propose a novel preventive therapeutic strategy aimed at achieving durable remission—or potentially a cure—in high‐risk patients with solid tumors. Central to this approach is immune manipulation, designed to be less susceptible to conventional drug resistance mechanisms. Importantly, resistance could be further mitigated by the brief duration and alternating nature of accompanying conventional therapies. We also highlight the utility of serum tumor marker LT as a unique experimental tool to monitor the progression of undetectable micrometastatic disease and calculate TDT. The proposed 7‐year treatment protocol aims to maintain a stable dormant state at micrometastatic niches, thereby enabling the “educated” immune system to autonomously eliminate any emergent proliferative foci over time.

In conclusion, this review critically evaluates the role and limitations of current targeted therapies in the most prevalent advanced solid tumors, while also addressing the biological and therapeutic complexity of MiRD. Based on both our own and others' experimental and clinical data, we present a novel treatment protocol offering high‐risk cancer patients an alternative and potentially curative strategy. This protocol is intended to be validated in future prospective, randomized clinical trials.

## Author Contributions

A.N. conceived, wrote, and revised the review article. P.F. wrote and revised. R.S. revised. D.B. wrote and revised. All authors have read and approved the final manuscript.

## Conflicts of Interest

The authors declare no conflicts of interest.

## Ethics Statement

The authors have nothing to report.

## Supporting information



Supplementary File 1: mco270373‐sup‐0001‐SuppMat.pdf

## Data Availability

The data that support the findings of this study are available from the corresponding author upon reasonable request.
