# Supplementary material for: Targeted Therapies in the Most Common Advanced Solid Tumors, Drug Resistance, and Counteracting Progressive Micrometastatic Disease: The Next Frontier of Research
Source: MedComm (2020). 2025 Sep 27;6(10):e70373. doi: 10.1002/mco2.70373 (PMC12475977; doi:10.1002/mco2.70373)
Supplement: Supplementary file 1 — mco270373‐sup‐0001‐SuppMat.pdf [file MCO2-6-e70373-s001.pdf]

Targeted therapies in the most common advanced solid tumours, drug resistance, and counteracting progressive micro-metastatic disease: the next frontier of research

Supplementary material

Andrea Nicolini[1], Paola Ferrari[2], Roberto Silvestri[3], and Dario A. Bini[4]

[1] Department of Oncology and New Technologies in Medicine, University of Pisa, Italy; andrea.nicolini@med.unipi.it
[2] Unit of Oncology 1, Azienda Ospedaliera Universitaria Pisana, Pisa, Italy; p.ferrari@ao-pisa.toscana.it
[3]Department of Biology, Genetic Unit, University of Pisa, Italy; roberto.silvestri@student.unipi.it
[4]Department of Mathematics, University of Pisa, Italy; dario.bini@unipi.it

August 12, 2025

1 Introduction

In this supplementary part, we report details about the analysis of the values at time t of a serological marker $m(t)$, and of the estimated volume $v(t)$ of the cancer that it may detect. This analysis, which relies on a mathematical model based on the exponential function, allows us to estimate the tumor doubling time (TDT), and the time t_1 the serological data reaches the cut-off value. If the size of the tumor is available at a given time t_2, say, by instrumental analysis like TAC, PET or ECO, then the model provides estimates of the size of the tumor at positive marker time together with the time t_0 the tumor started its doubling process.

This model, with the consequent estimates, is applied and verified on the clinical data of a set of patients affected by different kind of cancer.

In almost all the data analyzed, this study shows a sharp estimate of the cancer growth.

2 The mathematical model

We rely on the following assumptions:

1. The value of the serological marker $m(t)$ at time t is proportional to the volume $v(t)$ of the tumor at time t if the former is greater than or equal to the cut-off value. That is,

$$v(t) = \gamma m(t), \quad \text{for } m(t) \geq \text{cut-off},$$

   for a positive constant γ. If $m(t)$ is less than the cut-off, we assume that the specific value of $m(t)$ brings no information.

2. The volume $v(t)$ of tumor cells is exponential in time, as well as the value $m(t)$ of the marker if the latter is greater than or equal to the cut-off. That is, we assume that

$$m(t) = \alpha e^{\lambda t}, \quad \text{for } \; m(t) \geq \text{cut-off},$$

   for suitable positive constants α and λ.

3. The minimum size of the cancer detectable by the serological test, denoted by MS, is $MS = 3.5$mm. Therefore, the minimum volume detectable is $MV = MS^3$ mm^3. Actually, the value of MS may be different from case to case, generally it is in the range 2 – 7mm.

4. The minimum size detectable by TAC or Eco is $\widehat{MS} = 7$mm. Therefore the minimum volume detectable by TAC or Eco is 7^3mm^3.

We have the following properties in the case of an exponentional growth of a tumor.

a) The number of doublings for a tumor to pass from volume V_1 to volume V_2 is $\log_2(V_2/V_1)$.

b) If a tumor has volume V_1 at time t_1 and volume V_2 at time t_2, then the Tumor Doubling Time is TDT=$(t_2 - t_1)/\log_2(V_2/V_1)$.

c) Denoting V_0 the volume of a tumor when it starts its exponential growth at time t_0, and V_1 the known volume of the tumor at time t_1, then the value $t_1 - t_0$ is given by $t_1 - t_0 = TDT \log_2(V_1/V_0)$.

Crucial quantities that we will try to estimate in our analysis are

- TDT the tumor doubling time, given in days;
- CT the Cut-off time, that is the time when the serological marker reaches the cut-off value.

The TDT can be estimated relying on the exponential model by means of the expression

$$TDT = \frac{\log 2}{\lambda}. \tag{1}$$

This quantity provides an average estimate of the growth of the marker based on the log least-squares approximation.

A different estimate relies on the steepest incremental step Δ of the serological test by means of the formula

$$TDT = \frac{\log 2}{\Delta}, \quad \Delta = \max_i \frac{\log(m(t_i)) - \log(m(t_{i-1}))}{t_n - t_{i-1}} \tag{2}$$

where t_i is the time of the generic test where the marker is above the cut-off. This estimate relies on the maximum detected growth of the marker value.

A more balanced estimate that takes into account the global dynamic of the marker history together with its maximum variation is given by the arithmetic mean of the two latter quantities, namely,

$$TDT = \frac{\log(2)}{2}(\lambda^{-1} + \Delta^{-1}). \tag{3}$$

If volumes V_1 and V_2 of the tumor are known at two different times t_1 and t_2, respectively, then the TDT can be estimated by formula in item b) in the above list.

2.1 Estimating the parameters of the exponential model

From assumptions 1 we have $\gamma = v(t)/m(t)$, so that $\gamma = V_1/\text{cut-off}$, where V_1 is the volume of the tumor at the time when the marker coincides with the cut-off.

Under the above assumptions, the values of the parameters α and λ can be estimated by means of a linear least square minimization of the logarithm of the serological values.

More precisely, let t_i, y_i be the time and the value of the ith serological test, respectively, for $i = 1, 2, \ldots, n$, where n is the number of performed tests. We look for values of α and λ that minimize the log least-squares error

$$\sum_{i=1}^{N} (\log \alpha + \lambda t_i - \log y_i)^2.$$

The computation can be performed in Matlab [1] relying on the Singular Value Decomposition (SVD) [2].

Once the values of α and λ have been estimated, we may compute an estimate of the TDT by means of (3).

Together with the TDT, we may estimate other two values, namely,

- the cut-off time CT when the marker reaches the cut-off value, that is, $m(CT)$ =cut-off; this value is given by

$$CT = \frac{1}{\lambda} \log \frac{\text{cut-off}}{\alpha};$$

- the start time ST of the tumor given by the time the first doubling of the cells happened, i.e., such that $v(ST) = V_0$, given by

$$ST = \frac{1}{\lambda} \log \frac{V_0}{\gamma\alpha},$$

where V_0 is the estimated volume a tumor must have before it starts doubling.

3 Applying the mathematical model to clinical data

In this section, we perform the analysis of a set of clinical data by computing the estimated values of the constants α, λ, γ defining the model presented in Section 2 together with the values of the quantities TDT, CT and the lead time LT. More specifically we denote by TDT_{lsq}, TDT_{mxs}, the values of TDT estimated by means of (1) and (2), respectively, we denote TDT_{av} the arithmetic mean of the latter values computed by means of (3).

In the case where the instrumental clinical tests, say TAC or Eco, have been performed and the tumor size of volume V_2 has been estimated at time t_2, we provide also the estimate of TDT by means of equation in item b) of Section 1 using $V_1 = 3.5^3\text{mm}^3$, where t_2 is the time when the TAC or Eco has been performed, and $t_1 = CT$. We denote this latter estimate, performed at diagnosis time, by TDT_{dgn}.

We compute two estimates of the Lead Time, namely, LT_{co} is the LT with respect to the cut-off time CT, while LT_d is the LT with respect to the time DT (detect time) of the first positive marker test.

Three estimates of the Start Time are computed, that is the time when the tumor started its doubling process. They are based on TDT_{lsq}, TDT_{av}, TDT_{dgn} and are denoted by ST_{lsq}, ST_{av}, ST_{dgn}, respectively. Finally, the size Sz of the tumor at CT time is determined by relying on the instrumental determination of the size of the tumor, if available.

3.1 Clinical data

The data concern 10 patients and 11 recurrences that we subdivide according to the cancer type. Patients from P01 to P06 are affected by breast cancer, Patients P07, P08 by prostate cancer, Patient P09 (two recurrences P09, P9a) is affected by ovarian cancer and patient P10 by colon-rectal cancer.

Table S1 reports the values of all the parameters obtained by this analysis, while the remaining tables enhance some specific values.

Figure S1 reports, in logarithmic scale, the graph of the serological marker (blue color), where the values are enhanced by a "*", together with the graph of the exponential function (red color), that better approximates the data in terms of the logarithmic least squares. The figures display a horizontal straight-line,

Patient	TDT				CT	LT		ST			Size	Error
	lsq	mxs	av	dgn		co	d	lsq	av	dgn		
P01	272	75	174	192	-147	576	429	-1625	-1089	-1189	3.25	4.5e-02
P02	257	109	183	161	-104	641	537	-1497	-1096	-978	3.96	5.7e-02
P03	75	63	69	100	-25	402	377	-434	-401	-567	2.21	2.2e-02
P04	283	281	282	85	-153	387	234	-1688	-1683	-616	7.28	5.4e-06
P05	190	107	148	14	-6	90	84	-1034	-810	-83	13.04	6.9e-03
P06	37	9	23	23	-16	142	126	-218	-142	-138	3.65	1.1e+00
P07	209	63	136	40	-23	351	328	-1156	-760	-240	14.59	1.9e-01
P08	126	54	90	101	-31	461	430	-714	-520	-580	3.08	3.2e-01
P09	72	72	72	9	-76	83	7	-464	-464	-124	22.95	6.7e-30
P9a	207	137	172	87	-31	310	279	-1154	-963	-502	5.27	1.4e-02
P10	104	61	82	11	-92	121	29	-656	-539	-155	28.49	6.0e-03

Table S1: Values of all the estimated parameters. The TDT is estimated in different ways: TDT_{lsq} is based on the log least squares approximation, TDT_{mxs} is based on the maximum slope, TDT_{av} is their arithmetic mean, the value TDT_{dgn} is obtained by assuming size 3.5mm at cut-off time and relying on the size estimated at the instrumental diagnosis. The cut-off time CT is estimated in days before the detect time (assumed zero). The lead time LT is given with respect to the cut-off time (LT_{co}), and to the detect time (LT_{d}). The start time of the tumor is estimated in terms of the log least-squares approximation (ST_{lsq}), to the avergae estimate (ST_{av}), and to the instrumental diagnosis (ST_{dgn}) assuming size 3.5mm at cut-off time. The column "Size" displays the size of the tumor at cut-off time assuming a TDT equal to TDT_{av}. Finally, the column "Error" reports the least square errors in the log least-squares approximation.

in green color, that corresponds to the value of the cut-off. The data concern patients affected by breast cancer.

Figure S2 displays analogous plots for other 5 patients affected by prostate cancer (first line), ovarian cancer (second line), and colon-rectal cancer (last line).

3.2 Discussion

The first interesting observation is that in all the 11 cases displayed in the figures, the log-plots of the markers have an almost linear shape. This confirms that the exponential model is well suited to deal with the analysis of the marker values. This fact is also confirmed by the least-square errors, in the last column of Table S1, that are always less than 1 except for patient P06 where the error is slightly greater than 1.

A second remark, that stems from reading Table S1, is that for the patients affected by breast cancer (P01–P06), the estimates of the TDT given by the marker analysis and the instrumental analysis have a satisfactory matching in four cases out of six. In these cases, the estimated size of the tumor at cut-off time is close to the volume 3.5^3 mm^3 that we had assumed (compare with the column "Size" in Table S1).

The most evident mismatch concerns Patient P05 where the ratio between the TDT estimated by the marker and by the TAC is greater than 10. This fact can be explained observing that the size of the tumor at the cut-off time was already around 13mm diameter (see again column "Size"). This assumption is reasonable in view of the fact that the lead time is short with respect to the other cases.

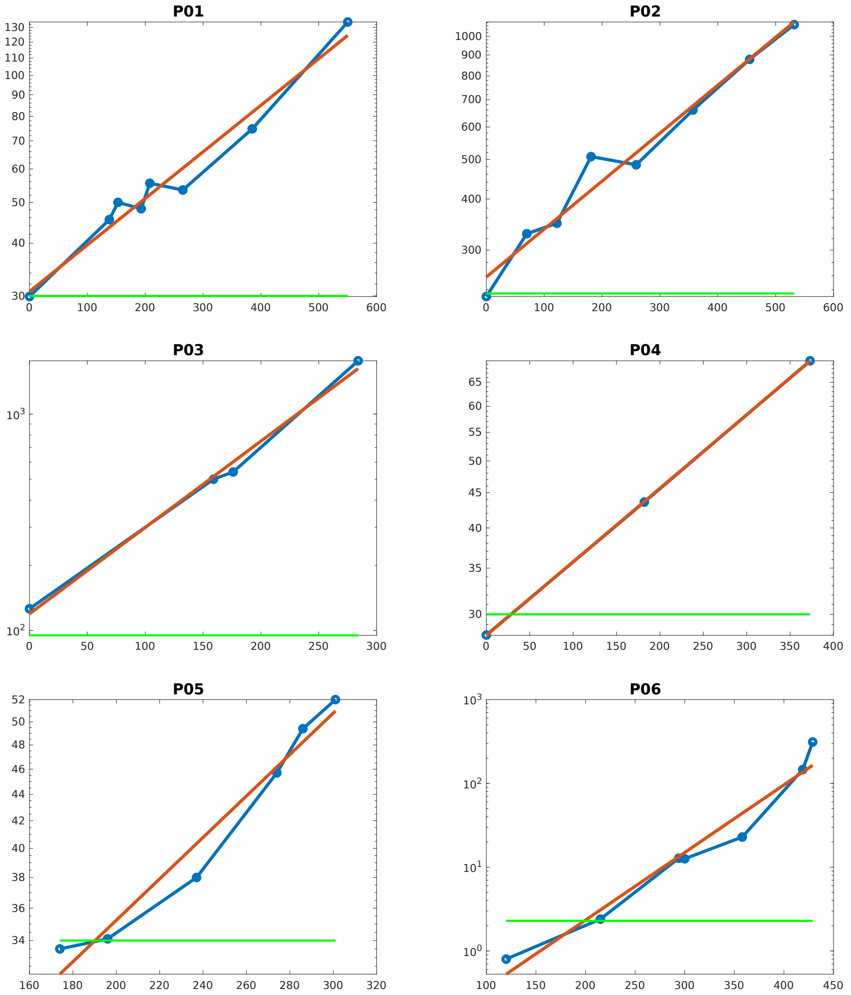

Figure S1: Log-scale plot of the markers (in blue) and of the approximating exponential function (in red). In the x-axis the number of days elapsed from the first serological tests, in the y-axis the value of the marker. The horizontal green line represents the value of the cut-off. The data of 6 patients affected by breast cancer are reported.

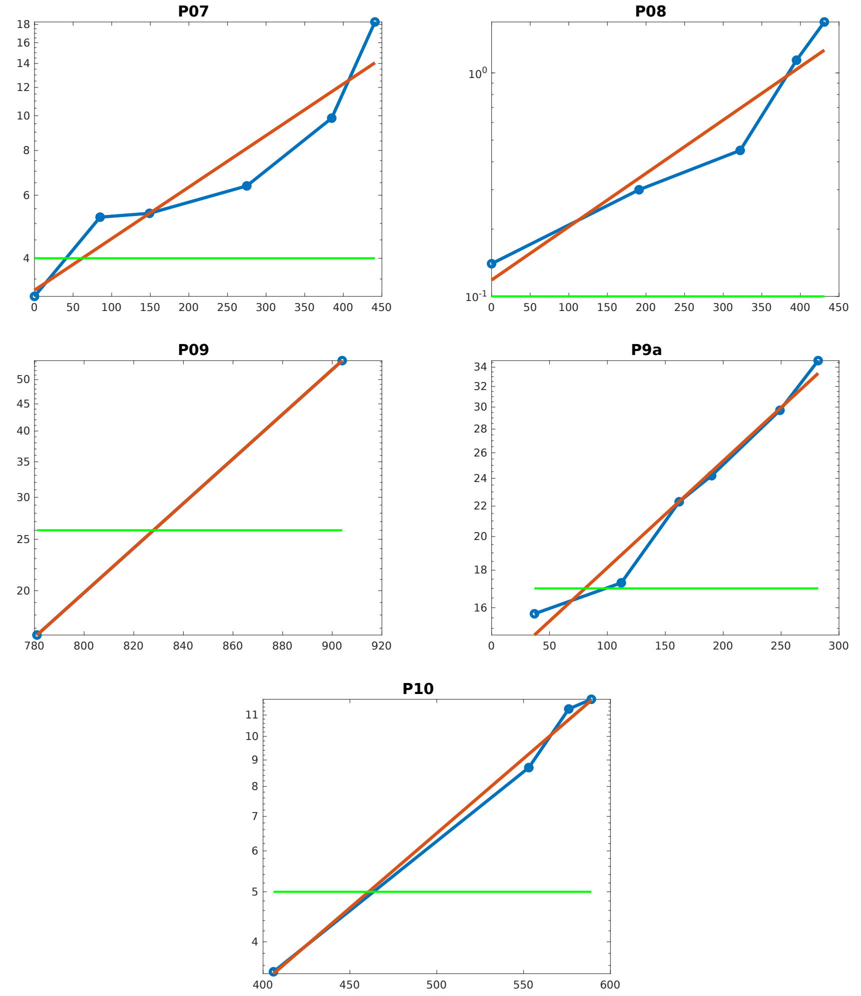

Figure S2: Log-scale plot of the markers (in blue) and of the approximating exponential function (in red). In the x-axis the number of days elapsed from the first serological tests, in the y-axis the value of the marker. The horizontal green line represents the value of the cut-off. The patients in lines 1,2, and 3, are affected by prostate, ovarian, and colon-rectal cancers, respectively.

	TDT_{av}	LT_d
median	136 (4.5)	279 (9.3)
mean	130 (4.3)	260 (8.7)

Table S2: Median and mean of the estimated values of the paramter TDT_{av} and LT_d out of 11 patients. Values are in days and in months (between parentheses).

Also for Patient P04 there is a slight mismatch in the estimates of TDT. Looking at the corresponding figure, and at the corresponding entry in the column "Error" of Table S1, we may see a clear exponential growth of the marker (straight line in the log-plot, and small corresponding least-squares error) the apparent mismatch actually informs us that the size of the tumor at cut-off time was about 7mm. In all the other 4 cases, the estimates of the size of the tumor at cut-off time are in the range [2.21,3.96] as expected from the serological test.

Similar remarks can be done in the other cases. We observe that the mismatch for Patient P09 is almost meaningless since the analysis is based on just two marker values, moreover, the LT is the shortest one among all the tested cases.

The medians and the mean values of the estimated parameters can be easily evaluated and are reported, in days (in months between parentheses) in Table S2.

A last remark concerns the tumor starting time. According to our model, the tumor started to grow from 5 months before the positive marker test, to about 4.6 years, with a mean of 2.1 years and a median of 2.08 years. It should be observed that this estimate relies on the following two assumptions: the minimum size at which a tumor starts doubling is estimated 1mm; the TDT remains constant at each stage of the tumor growth. Concerning the latter assumption, it has been observed in the literature that in the earlier moments of the tumor life, the growth is faster than in the subsequent times. This means that the estimates given above of the tumor start time should be modified accordingly.

4 The Matlab code

The listing of the Matlab functions used for performing the data analysis can be provided upon request from the authors.

References

[1] The MathWorks Inc. (2022). MATLAB Version: 9.11.0.1769968 (R2021b) Natick, Massachusetts: The MathWorks Inc. https://www.mathworks.com

[2] G.H. Golub and C. Van Loan, *Matrix Computations.* Johns Hopkins University Press, 2013,
